# Network modelling reveals the mechanism underlying colitis-associated colon cancer and identifies novel combinatorial anti-cancer targets

**DOI:** 10.1038/srep14739

**Published:** 2015-10-08

**Authors:** Junyan Lu, Hanlin Zeng, Zhongjie Liang, Limin Chen, Liyi Zhang, Hao Zhang, Hong Liu, Hualiang Jiang, Bairong Shen, Ming Huang, Meiyu Geng, Sarah Spiegel, Cheng Luo

**Affiliations:** 1 State Key Laboratory of Drug Research, Shanghai Institute of Materia Medica, Chinese Academy of Sciences, Shanghai, China; 2 Soochow University, Center for Systems Biology, Jiangsu, China; 3 Department of Biochemistry and Molecular Biology, Virginia Commonwealth University School of Medicine, Richmond, VA 23298, USA

## Abstract

The connection between inflammation and tumourigenesis has been well established. However, the detailed molecular mechanism underlying inflammation-associated tumourigenesis remains unknown because this process involves a complex interplay between immune microenvironments and epithelial cells. To obtain a more systematic understanding of inflammation-associated tumourigenesis as well as to identify novel therapeutic approaches, we constructed a knowledge-based network describing the development of colitis-associated colon cancer (CAC) by integrating the extracellular microenvironment and intracellular signalling pathways. Dynamic simulations of the CAC network revealed a core network module, including P53, MDM2, and AKT, that may govern the malignant transformation of colon epithelial cells in a pro-tumor inflammatory microenvironment. Furthermore, *in silico* mutation studies and experimental validations led to a novel finding that concurrently targeting ceramide and PI3K/AKT pathway by chemical probes or marketed drugs achieves synergistic anti-cancer effects. Overall, our network model can guide further mechanistic studies on CAC and provide new insights into the design of combinatorial cancer therapies in a rational manner.

Inflammation and cancer are closely correlated^1^. The link between inflammation and cancer development is especially strong in patients with colorectal cancer (CRC), which is one of the most common malignancies and a leading cause of cancer mortality worldwide^2^. An increased risk of CRC development has been observed in patients with inflammatory bowel disease (IBD)^3^, and nonsteroidal anti-inflammatory drugs are effective in preventing colon neoplasia^4^. Dysregulations of the immune microenvironment and several inflammation-related signalling pathways, such as TNF-α/NF-κB, IL-6/STAT3, COX-2/PGE_2_ and TGF-β/SMADs, have been shown to contribute to the development of inflammation-associated cancers^5^,^6^,^7^,^8^,^9^. In addition, emerging evidence suggests a possible link between the inflammatory microenvironment and cancer therapy resistance^10^. Nevertheless, most of these studies have focused on a single molecule or pathway. Information on how the immune microenvironment affects cancer development and how the inflammatory signalling pathways crosstalk with classical tumourigenesis pathways is still lacking. Therefore, to gain a holistic view on the mechanism of the development of inflammation-associated cancers, as well as to identify effective therapeutic targets, the extracellular microenvironment and intracellular signalling should be considered as a complex system and studied in a more systematic manner.

To date, network modelling has been successfully used in the study of complex biological systems^11^,^12^,^13^. Existing knowledge of individual pathways can be incorporated into an integrated biological network, which could be further converted into a dynamic and predictive model using various mathematical modelling techniques. Boolean network models are the simplest discrete mathematical models and assume only two states (ON or OFF) for each node in the biological networks. Dynamic Boolean network models have been successfully applied in studies of complex diseases and biological processes, such as survival signalling of T-cell large granular lymphocyte (T-LGL) leukaemia^13^, hepatocyte growth factor (HGF)-induced keratinocyte migration^12^, immune cell differentiation^14^, and cell cycle regulation^11^. Boolean network models have also been used to integrate microenvironment components and signaling pathways to study cancer biology and predict therapy outcomes^15^,^16^. Boolean network models are especially useful when the biochemical kinetic parameters of a certain biological process are unknown or the networks contain different species of biological entities, such as proteins, small molecules, mRNAs, and even cells.

In the present work, we constructed a Boolean network model describing the growth and survival of preneoplastic epithelial cells in an inflammatory microenvironment, aiming to systematically study the molecular mechanisms underlying the development of colitis-associated colon cancer (CAC). The ability of the network model to recapture experimental observations validated its rationality. The detailed dynamic properties of the CAC network model under normal or dysregulated inflammatory microenvironments were characterised. Our simulation results suggest the constant activation of the node representing dendritic cells (DC) creates a pro-tumor inflammatory microenvironment. Attractor analysis identified a key regulatory module involving P53, MDM2, GSK3-β and AKT signalling that may govern the malignant transformation of epithelial cells in this pro-tumour inflammatory microenvironment. Furthermore, *in silico* perturbation studies and experimental validations led us to identify several novel drug combinations that could significantly inhibit proliferation and induce apoptosis of tumour cells under an inflammatory stimulus. Taken together, our study integrates the extracellular microenvironment and intracellular signalling to provide a holistic view of inflammation-associated cancer. Our dry lab model and experimental findings can accelerate mechanistic studies and the development of novel combinatorial therapies for CAC and other inflammation-associated cancers.

## Results

### The CAC network representing intestinal epithelial cells in an immune microenvironment

**Figure 1:**
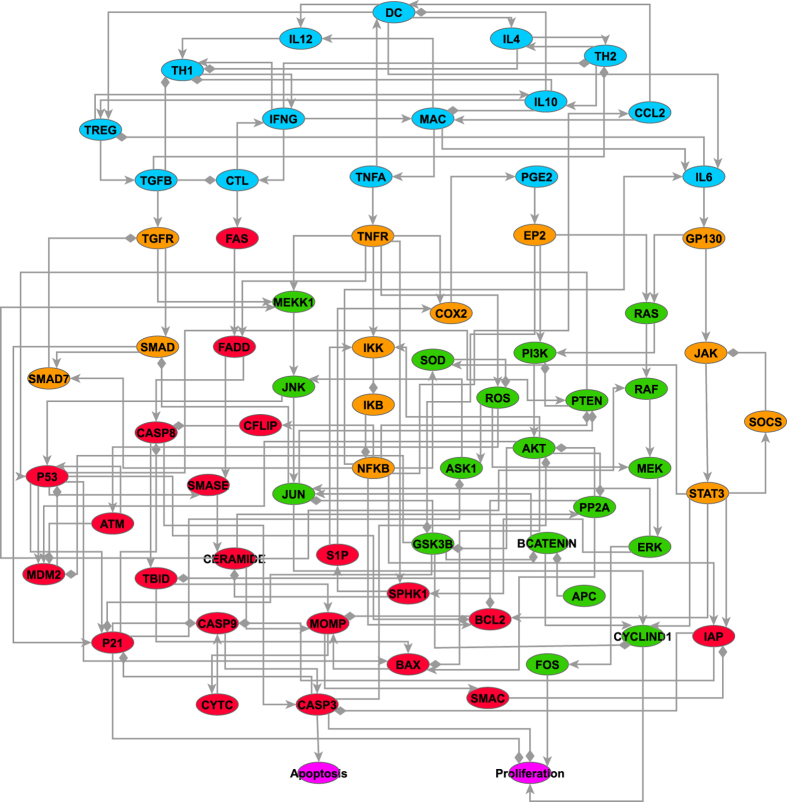
Topology of the CAC network. Five colours were used to represent the nodes with different biological functions. The nodes in cyan belong to the extracellular immune microenvironment; the nodes in orange primarily participate in inflammatory signalling; the nodes in green primarily mediate cell proliferation; and the nodes in red regulate cell survival. The two purple nodes represent the output effects (proliferation and apoptosis) of the network model. An arrowhead represents positive regulation (activation or upregulation), whereas a diamond indicates negative regulation (inhibition or downregulation).

By performing extensive literature and database searches, we constructed a knowledge-based network linking inflammatory signalling and cell proliferation and survival pathways of premalignant intestinal epithelial cells (IECs) (Fig. 1). We designated this network model as the CAC network. The entire CAC network incorporates 70 nodes and 153 edges. It can be divided into two parts: the IEC part, which contains nodes representing intracellular signalling components, and the immune microenvironment part, which contains the nodes representing immune cells, cytokines and chemokines. We also modelled ‘Proliferation’ and ‘Apoptosis’ as two output nodes to summarise the final biological effects of the inflammatory signalling. The nomenclature of all the nodes in the network is provided in Supplementary Table S1, and the biological description of the CAC network is presented in Supplementary Methods. The topology properties of the CAC network are summarised in Supplementary Table S2. The properties of the CAC network resemble those of general biological networks, which are characterised by higher clustering coefficient than random networks (Supplementary Table S2) and approximate power-law distributions of node degrees (Supplementary Fig. S1). However, as the size of the CAC network is relatively small and its topology has been simplified, the quantitative characterisation of the network topology is not very informative and therefore we are focusing more on the dynamic properties of the CAC network.

To further characterise the dynamic cell signaling events, we translated the CAC network into a Boolean network model, in which the network node was described by one of two possible states: ON or OFF. The ON state can be biologically interpreted as the activation of a gene/protein, or the production of a small molecule whereas the OFF state means the inhibition of a gene/protein or the absence of a small molecule. The regulatory relationships between upstream nodes (regulators) and downstream nodes (targets) are expressed by the logical operators AND, OR and NOT. The Boolean logical rules that govern the states of all these nodes are listed in Supplementary Table S3 and a thorough justification of these rules is provided in Supplementary Methods. Preliminary robustness tests suggested the CAC network model was robust to small amounts of noise (Supplementary Fig. S2), which is in accord with the general feature of biological networks.

### Dynamics of the CAC network model in a normal immune microenvironment

**Figure 2:**
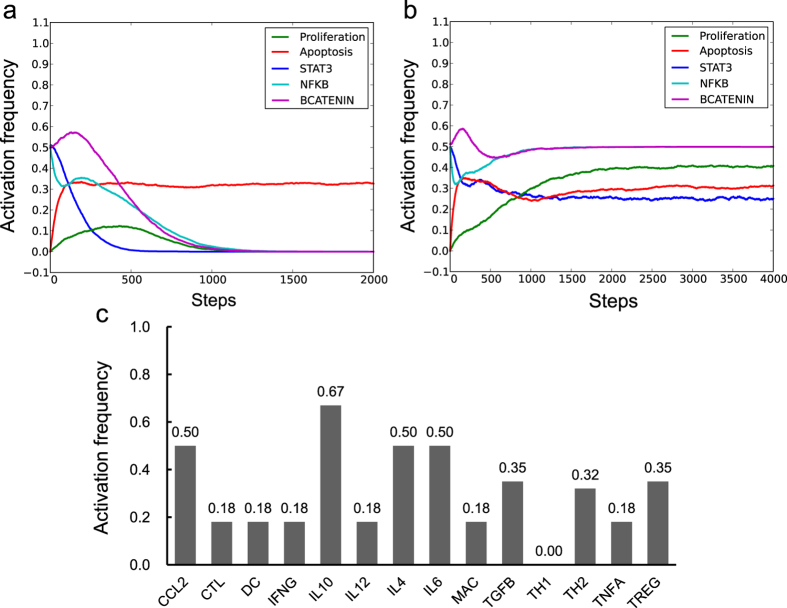
Dynamics of the CAC network model in the non-inflammatory microenvironment. (**a**,**b**) Activation frequencies for five nodes, including Proliferation, Apoptosis, STAT3, NFKB and BCATENIN, were observed in the non-inflammatory microenvironment (**a**) and during transient activation of the DC node (**b**,**c**) The stabilised activation frequencies for all the microenvironment nodes when the initial state of DC was set to ON and other microenvironment nodes were initially set to OFF.

We first examined whether our CAC network model could reproduce the experimental observations of the IECs in a normal immune microenvironment, including a non-inflammatory microenvironment and a normal inflammatory response. To simulate a non-inflammatory microenvironment, we fixed the states of all the nodes in the immune microenvironment (cyan nodes in Fig. 1) to OFF to represent the absence of inflammatory factors. We also fixed the state of the APC node to ON to represent premalignant IECs, in which the adenomatous polyposis coli (APC) protein is constantly expressed and activated to suppress β-catenin signalling^17^. Subsequently, we iterated the model using the general asynchronous (GA) updating method from a large number (5,000) of randomly selected initial states. When simulating a dynamic Boolean model using asynchronous updating methods, the frequency of a node being in the ON state (activation frequency) can give qualitative indications of the probability that a certain signaling component or a biological process being activated in a real cell. The activation frequencies of the two output nodes – Proliferation and Apoptosis at each simulation step were recorded to evaluate the impact of the microenvironment on cell survival states (Fig. 2a). In order to facilitate the comparison between our simulation results and previous experimental observations, we also recorded the activation frequencies of three nodes representing STAT3, NF-κB and β-catenin transcription factors (STAT3, NFKB and BCATENIN), whose activations have been considered as hallmarks of CAC^17^,^18^,^19^,^20^. After 1000 steps of iteration, the Proliferation node rested in the OFF state, suggesting the IECs were unable to proliferate under non-inflammatory microenvironment. STAT3, NFKB and BCATENIN also stabilised in the OFF state within 1500 steps (Fig. 2a). By contrast, the steady activation frequency of the Apoptosis node was approximately 30%, indicating that a fraction of the epithelial cells could undergo apoptosis under non-inflammatory conditions. We then performed network reduction of the CAC network under the non-inflammatory microenvironment and identified its attractors (Supplementary Table S4). Attractors present the long-term behaviours of a Boolean model and can be regarded as potential stable states of a cell under certain conditions^21^. In consistence with the numerical simulation results from random initial states, two attractors, which represents the resting and apoptosis states of epithelial cells can be identified under this condition. These simulation results can be biologically interpreted as the tendency of IECs to remain in a resting state without inflammatory signals, but they also possess the capability to undergo spontaneous apoptosis. Above simulation results are supported by previous findings that spontaneous apoptosis of the colon epithelium is a crucial mechanism for maintaining the homeostasis of gastrointestinal tissues^22^.

Dendritic cells (DCs) are known as the most potent dedicated antigen-presenting cells specialised to initiate and maintain immunity and tolerance^23^. Therefore, we next simulated the normal initiation of an inflammatory response by setting the initial state of DC to the ON state to simulate the transient activation of dendritic cells. Compared with the simulation results in the non-inflammatory microenvironment (Fig. 2a), the transient activation of DC moderately increased the activation frequency of Proliferation (Fig. 2b), whereas the activation frequency of Apoptosis was unchanged. In accordance, the CAC network under this condition possessed attractors that represent proliferation phenotypes in addition to apoptosis and resting phenotypes (Supplementary Table S4).The transient activation of DC also activated STAT3, NFKB and BCATENIN, as well as other immune cells with different activation frequencies; however, none of them exceeded 0.5 (Fig. 2c and Supplementary Fig. S3). The activation frequencies of the immune suppressive nodes, such as IL10, was generally higher than that of the pro-inflammatory nodes, such as TNFA and IL6 (Fig. 2c). Therefore, our model suggests that the transient activation of DCs may initiate a controlled inflammatory reaction and eventually lead to an immune suppressive microenvironment, which does not support the uncontrolled growth of epithelial cells. These results are in agreement with observations that the normal microenvironment of colon mucosa is in an immune suppressive state, even if the colon contains a large amount of microbiota and antigens^24^.

### Effect of different immune microenvironments on IECs

Because the inflammatory microenvironment is a mixture of different types of infiltrated immune cells, we evaluated the effect of different immune microenvironments on IECs by iteratively fixing one or a group of immune cell nodes at the ON state to mimic their constant presence in the microenvironment. The activation frequencies of Proliferation, Apoptosis, and different cytokine nodes in the corresponding immune microenvironment are shown in Table 1. Because there were 63 different combinations, only the combined activations of immune cell nodes that have additive effects to single activations are shown. We observed that in contrast to the transient activation of DC, maintaining DC in the ON state permanently, which could be biologically interpreted as the constant activation of dendritic cells, created the most pro-proliferation microenvironment, in which the activation of Proliferation significantly increased and the activation of Apoptosis was blocked (line 1). This observation is supported by experimental findings that although the transient activation of DCs initiates a controlled inflammatory reaction^24^, the sustained activation of DCs leads to chronic inflammation in IBDs^25^, which enhances the growth and survival of IECs^7^,^26^. In addition to DC, the constant activation of MAC, CTL or TH1 in the microenvironment could also increase the activation of Proliferation while decreasing the activation of Apoptosis (lines 2, 4 and 5).

However, constant activation of TREG or TH2 slightly induced the activation of Proliferation but significantly increased the activation of Apoptosis (lines 3 and 6). Upon TREG activation, we observed increased activation of IL10 and TGFB and decreased activation of TNFA and IL6 (line 3 compared with lines 1 and 2). This observation is consistent with previous findings in which regulatory T cells were shown to reduce tumour growth in CAC cases by producing immune suppressive cytokines (e.g., IL-10 and TGF-β) and reducing pro-inflammatory cytokines (e.g., TNF-α and IL-6)^27^. Interestingly, although the activation of CTL alone induced the activation of Proliferation but not Apoptosis (line 4), the combined activation of CTL and TREG significantly reduced Proliferation and increased Apoptosis, forming the most anti-tumourigenic microenvironment (line 11). This result reiterates the clinical phenomenon that CTL contributes to intestinal inflammation and promotes tumour growth in CAC cases^28^, despite of previous observations that infiltration of CTL is commonly correlated with favourable prognosis in sporadic colon cancers^29^. The effect of combined activation of CTL and TREG on the states of Apoptosis indicates that the additional activation of Treg cells can restore the cytotoxic function of CTL and therefore enhance immune surveillance.

We also identified several novel immune cell combinations that exhibited various effects on the states of Proliferation, Apoptosis and the cytokine nodes (lines 7–10 and lines 12–14), revealing the complex influence of the immune microenvironment on the survival and proliferation of IECs. These predictions can be useful for rationally designing immune therapies to restore normal microenvironments or for building anti-tumour microenvironments by modulating immune cells.

### Dynamics of the CAC network model in a pro-tumour inflammatory microenvironment

**Figure 3:**
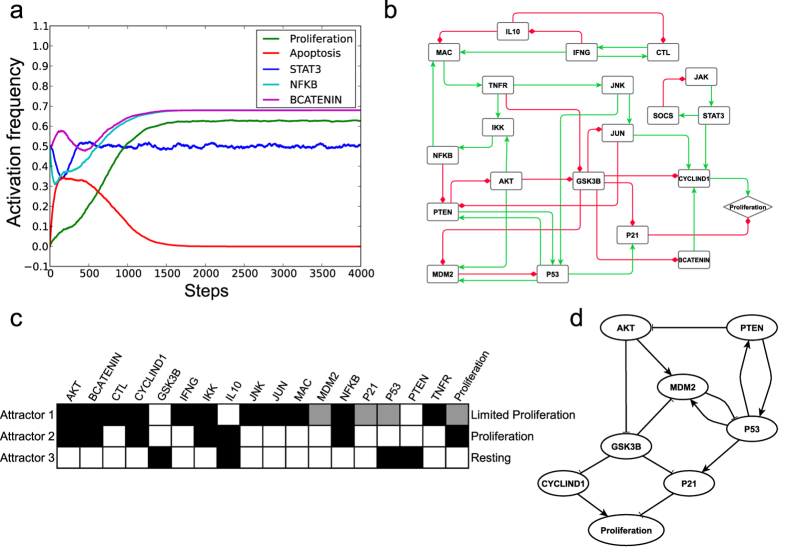
Dynamics of the CAC network model in the pro-tumour inflammatory microenvironment. (**a**) The activation frequencies of Proliferation, Apoptosis, STAT3, NFKB and BCATENIN were observed in the pro-tumour inflammatory microenvironment. (**b**) The final reduced CAC network topology in the pro-tumour microenvironment. A green line with an arrowhead represents positive regulation, whereas a red line with a diamond indicates negative regulation. (**c**) The node activation patterns in the 21-node sub-network. Only the nodes that possess different activation patterns in the three attractors are shown. White, grey and black boxes represent inactivation, partial activation and full activation, respectively. (**d**) The core regulatory network that governed the behaviour of the CAC network in the pro-tumour inflammatory microenvironment.

Notably, Proliferation did not reach full activation, even under the strongest tumour-promoting microenvironment (fixing DC at ON). This result could be biologically interpreted as a controlled growth of the premalignant IECs in an inflammatory microenvironment. We then characterised the dynamic properties of the CAC network model under this tumour-promoting microenvironment to understand the regulatory mechanisms of cellular proliferation and to identify the factors responsible for the malignant transformation of the IECs. After a transient activation, Apoptosis was eventually stabilised in the OFF state in all simulations, whereas the activation frequency of Proliferation was approximately 0.6 (Fig. 3a). The activation frequencies of STAT3 and NFKB were significantly increased compared with those in the non-inflammatory condition and the normal inflammatory response (Fig. 3a compared with Fig. 2a,b), which is consistent with experimental observations that these two transcription factors were highly active under inflammatory stimulus^7^,^30^. BCATENIN was partially activated, although we maintained APC in the ON state. Fixing APC in the OFF state to simulate the inactivation mutation of APC protein led to the full activation of BCATENIN. However, the activation frequency of Proliferation was not further increased (Supplementary Fig. S4). Clinical observations have shown that although APC mutations are one of the earliest events in sporadic colorectal cancers and are considered essential for the transition of preneoplastic cells to aberrant crypt foci and adenoma^17^,^31^, these mutations occur much later in CAC cases^32^,^33^.

To further study the dynamic properties of the CAC network in the pro-tumor inflammatory microenvironment, we performed network reduction and analysed the attractor structure of the CAC network under this pro-tumour microenvironment (fixing DC in the ON state). The first step of network reduction identified 33 nodes that were stabilised either in the ON or OFF state. In particular, the nodes correlating with cell proliferation, such as RAS, RAF, MEK, ERK, and FOS, were stabilised in the ON state. Previous studies have shown that ERK/MAPK (extracellular signal-regulated kinase/mitogen-activated protein kinase) signaling can be activated by pro-inflammatory cytokines in IBD cases^34^ and that they are over-activated in both sporadic colon cancer and CAC^35^,^36^. In addition, Apoptosis and other nodes correlating with apoptotic cell death, such as BAX, TBID, CERAMIDE, CYTC, CASP3, CASP8, CASP9, MOMP and PP2A, were all stabilised in the OFF state, indicating the entire apoptosis pathway was blocked by the pro-tumor inflammatory microenvironment. After removing stabilised nodes, simple intermediate nodes, and nodes with zero out-degrees, the final reduced network comprised 21 nodes and 39 interactions (Fig. 3b). The Boolean rules governing the reduced network are provided in Supplementary Table S5. We identified three attractors of the reduced CAC network model: a complex attractor (Attractor 1) that contained 48 states, and two cyclic attractors (Attractors 2 and 3) that contained six states each (Table 2). By analysing the system’s states within these attractors, we found three activation patterns for each node in the sub-network. One pattern was full activation, in which the node was stabilised at ON in all states within an attractor; another pattern was partial activation, in which the node states oscillated between ON and OFF and half were ON; and the last pattern was inactivation, in which the node was stabilised at OFF in all states. Nodes STAT3, JAK and SOCS formed a negative feedback loop, and they oscillated in all the attractors; however, their activation patterns were the same. Therefore, these three nodes were excluded from further activation pattern analysis. Figure 3c shows the different activation patterns of the nodes within the three attractors. The pro-proliferate nodes AKT, BCATENIN, CYCLIND1, IKK, JUN and NFKB were all fully activated in Attractor 1, indicating a tendency for the IECs to undergo proliferation. However, P53 and P21 were partially activated in Attractor 1. Thus, the activation frequency of Proliferation was then restricted by the partial activation of P21 according to the Boolean function (Proliferation* = CYCLIND1 and not P21) of the reduced CAC network. Therefore, Attractor 1 represented a limited proliferation state. In Attractor 2, AKT, BCATENIN and CYCLIND1 were fully activated, and P21 was inactivated; thus, this attractor represented a proliferation state. Both CYCLIND1 and P21 were inactivated in Attractor 3; therefore, this attractor represented a resting state. When using synchronous updating methods, we found the reduced network had 28 attractors, which could also be grouped into three phenotypic classes: resting, limited proliferation and proliferation (Supplementary Table S6).

By iteratively fixing one node in the sub-network to either an ON or OFF state, we identified a core regulatory network that governed the behaviour of the CAC network in the pro-tumour environment (Fig. 3d). Fixing AKT or CYCLIND1 in the OFF state could cause all state trajectories to fall into the resting attractor. Fixing GSK3B, P21, P53 or PTEN in the ON state had the same effect on the attractor landscape. Currently, alteration of the PTEN/PI3K/AKT signalling axis is a well-accepted driving force in carcinogenesis, and AKT has been shown to be dysregulated in most colon cancers^37^,^38^. Notably, our model herein emphasised that AKT activation was essential for accessing the attractor representing proliferation under an inflammatory stimulus. In addition, our model suggested that GSK3-β might play a tumour suppressor role in CAC by inhibiting CYCLIND1 and MDM2 in the pro-tumor inflammatory microenvironment (Fig. 3d). Most importantly, we observed that only the permanent inactivation of P53 or the activation of an endogenous P53 inhibitor, MDM2, could lead all the simulation trajectories to fall into the proliferation attractor. This effect can be biologically interpreted as a mutation or the constant suppression of P53, endowing IECs with the capability of uncontrolled growth and thereby initiating malignant transformation. Therefore, the simulation results suggest P53 pathway may act as the last guard before malignant transition in a strong pro-tumour inflammatory microenvironment, which is supported by the observation that P53 mutation (but not APC mutation) is a common event in the initiation phase of CAC progress^39^. The changes of the output effects under various conditions showed similar trends when different updating methods were used (Supplementary Table S7), indicating the overall dynamic properties of our Boolean network are not sensitive to the updating methods.

### Key nodes regulating malignant transformation revealed by systematic node perturbations

We subsequently performed a systematic node perturbation analysis on the entire CAC network to identify other nodes that may mediate malignant transformation in an inflammatory microenvironment. We maintained DC in the ON state to mimic the premalignant IECs in a tumour-promoting microenvironment and perturbed the states of other nodes in the CAC network. For the nodes that became stabilised in either an ON or OFF state, we fixed each node in the opposite of its stabilised state and continued updating other nodes. For the oscillated nodes, we perturbed each node twice by fixing the node to either ON or OFF. These perturbations mimic the manipulation of biological systems through genetic or chemical approaches, such as gene knockdown or treating cells with active compounds. We then observed the stabilised activation frequencies of the output nodes, Proliferation and Apoptosis, to evaluate the perturbation effect. Through this method, we identified 36 of 109 perturbations that could affect the activation of Proliferation or Apoptosis. We manually categorised these perturbations into pro-proliferative, anti-proliferative, and pro-apoptosis groups according to their effects on the states of Proliferation and Apoptosis.

**Figure 4:**
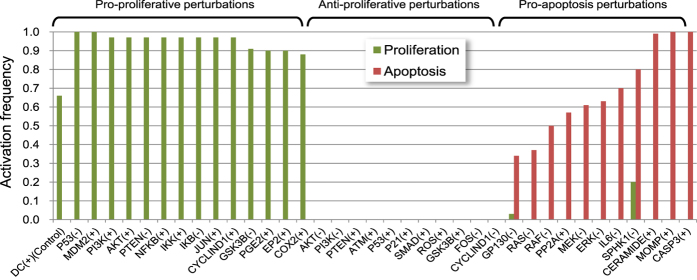
Node perturbation results of the CAC network model in the pre-transformed state. The activation frequencies of node Proliferation and Apoptosis corresponding to each perturbation are shown. (+) indicates that the node was fixed in the ON state, and (−) indicates that the node was fixed in the OFF state.

As shown in Fig. 4, the pro-proliferative group contains the perturbations that lead to a high activation frequency (>90%) of Proliferation. The perturbed nodes in this group come from four pathways: the P53 pathway (P53, MDM2), the PI3K/AKT pathway (PI3K, AKT, PTEN, and GSK3B), the NF-κB pathway (NFKB, IKK, IKB) and the COX2/PGE2 pathway (PGE2, EP2 and COX2). The P53 node and PI3K/AKT pathway nodes also exists in the anti-proliferative group when set in the opposite states (Fig. 4). The critical roles of P53, MDM2 and AKT in CAC progress have been revealed by the above attractor analysis. The aberrant activation of NF-κB and COX2/PGE2 signalling has also been detected in most CAC cases^7^,^9^. The pro-apoptotic perturbations include the inhibition of ERK MAPKs pathway nodes (RAS, RAF, MEK and ERK) and the inhibition of IL6 signalling nodes (IL6 and GP130). Previous findings indicate that ERK MAPKs are the major regulators of proliferation during colon carcinogenesis^35^, and the suppression of the ERK MAPK pathway inhibits proliferation and induce apoptosis of IECs in an inflammatory microenvironment^40^. Blocking IL-6 signalling with an anti-interleukin-6 receptor antibody or inhibiting IL-6 *trans*-signalling with TGF-β has also been shown to suppress tumour progression in colon cancer^41^. Notably, the perturbations of the nodes that participate in sphingolipid metabolism (CERAMIDE, SPHK1) are part of the pro-apoptotic group. A recent finding has demonstrated that SPHK1/S1P signalling plays a crucial role in linking chronic inflammation and CAC and that inhibiting SPHK1 could effectively reduce CAC development^42^.

In all, 29 of 36 predictions made by *in silico* perturbations can be supported by previous experimental observations, which validates the rationality of our model (Supplementary Table S8). The perturbation analysis also led to the novel prediction that activation of GSK3B, SMAD, ROS, PP2A, ATM, or CERAMIDE could inhibit proliferation or induce apoptosis of preneoplastic IECs in the pro-tumor inflammatory microenvironment, indicating that these molecules can be potential therapeutic targets for preventing CAC development.

### *In silico* double perturbation study and experimental validations identify novel drug combinations

As both our model and previous experimental studies indicate that P53 is crucial for the malignant transformation of IECs in an inflammatory microenvironment, we maintained DC in the ON state and P53 in the OFF state simultaneously to mimic the state of a neoplastically transformed epithelial cell in a pro-proliferative microenvironment. We subsequently performed perturbation analysis on the CAC network model under this condition to identify potential therapeutic targets for treatment of CAC. In this situation, only 18 of 89 perturbations could lower the activation frequency of Proliferation by over 50% (Supplementary Fig. S5), indicating that the CAC network in the neoplastically transformed state was more robust than that in the pre-transformed state. Only two perturbations could induce the activation of Apoptosis: activation of MOMP and activation of CASP3 (Supplementary Fig. S5). However, MOMP or Caspase3 activation represent the terminal events in apoptotic cell death, and therefore, manipulating these processes therapeutically is impractical. The robustness of the CAC network model in the P53-inactive state suggested that the single target therapy may be less effective in killing tumour cells that had previously developed in CAC cases. We further performed double perturbations by altering the state of two nodes simultaneously to seek possible combinatorial therapeutic approaches. We found several combined perturbations that could inhibit proliferation while increasing apoptosis (Table 3). However, not all of these perturbations are therapeutically accessible because of the lack of modulators, such as small molecule inhibitors. Among the double perturbations, we found some promising combinations that involve the activation of the CERAMIDE pathway while inhibiting the PI3K/AKT pathway. The above dynamic analysis indicated that AKT may play an important role in forming the attractor for the proliferation state, and several inhibitors of the PI3K/AKT pathway are under clinical evaluation^43^. Ceramide has also been previously shown to induce apoptosis and to sensitise tumour cells to radiotherapy^44^,^45^. Therefore, these combinations may be more clinically applicable than other predicted combinations.

**Figure 5:**
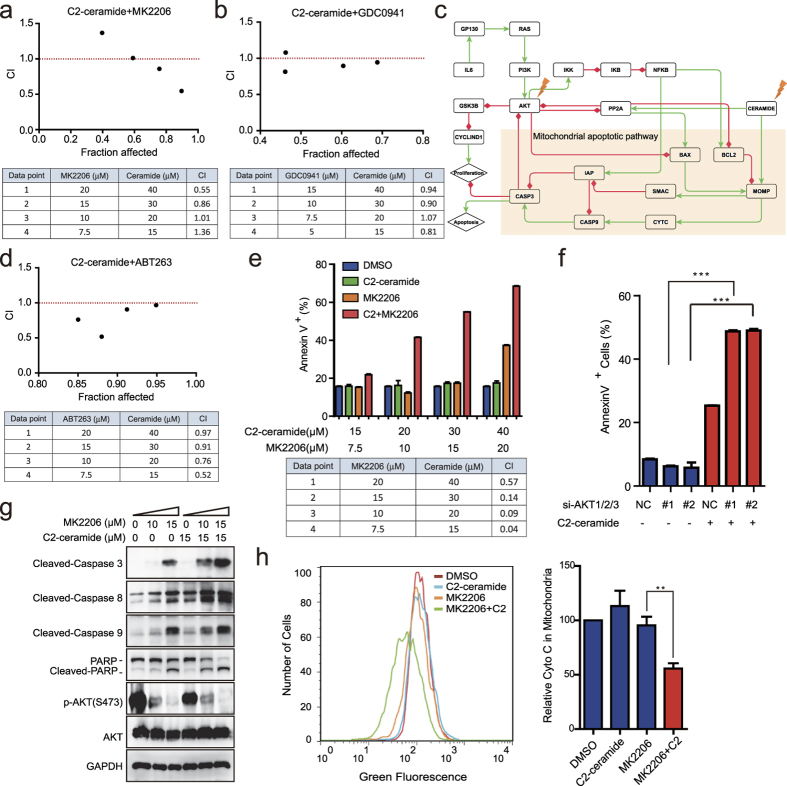
Experimental validations of drug combination predictions that targeting AKT plus exogenous C2-ceramide reduced cell viability and increased cell apoptosis. (**a**) The combination effects of C2-ceramide and MK2206 on the viability of HT29 cells were determined by calculating the CI values for each data point. CI < 1 indicates a synergistic effect. (**b**) The combination effects of C2-ceramide and GDC0941 on the viability of HT29 cells. (**c**) The sub-network related to PI3K/AKT and ceramide signalling extracted from the entire CAC network. A green line with an arrowhead indicates positive regulation (activation or upregulation), whereas a red line with a diamond indicates negative regulation (inhibition or downregulation). (**d**) The combination effects of C2-ceramide and ABT263 on the viability of HT29 cells. (**e**) Synergistic apoptotic effects of C2-ceramide and MK2206 on HT29 cells. (**f**) Mean percentage of apoptotic cells treated with siAKT1/2/3 and/or 15 μM C2-ceramide. ‘NC’ group stands for the scrambled negative siRNA pools, which was used as a negative control; ‘#1’ and ‘#2’ groups stand for AKT siRNA pools that contain different siRNA sequences listed in Supplementary Methods. (**g**) Immunoblots of lysates from cells treated with 15 μM C2-ceramide and 10 μM or 15 μM MK2206 for 24 h. (**h**) Flow cytometry detection of mitochondrial cytochrome C levels in HT29 cells treated with DMSO (red line), 15 μM C2-ceramide (blue line), 10 μM MK2206 (orange line) and 15 μM C2-ceramide+10 μM MK2206 (green line) for 24 h. The bar graphs (right) show the relative fluorescence intensities representing mitochondrial cytochrome C levels. Data are representative of three independent experiments (mean ± s.e.m.). **P < 0.01 and ***P < 0.001 (Student’s t-test). ns, not significant.

We then proceeded to validate the utility of these combinatorial perturbations in HT29 colon cancer cells, which have a P53 R273H inactivation mutation^46^. The impact of the inflammatory microenvironment was integrated by constantly exposing cells to the treatment with IL6 plus TNF-α, which resulted in the activation of STAT3 and NF-κB signalling (Supplementary Fig. S6a). As predicted, short chain, cell permeable C2-ceramide, which has been shown to increase endogenous long chain ceramides^47^, exerted synergistic effect with the AKT pan-inhibitor MK2206 (Fig. 5a) or PI3Kα/δ inhibitor GDC0941 (Fig. 5b) on cell viability under several concentrations, indicated by a combination index (CI) less than 1.

To further understand the mechanism of the combinational effect of AKT inhibitors and ceramide, we extracted the sub-network related to ceramide, the PI3K/AKT pathway and inflammatory activation from the global CAC network. According to the sub-network shown in Fig. 5c, PI3K/AKT and ceramide signalling converged on the mitochondrial apoptotic pathway. Ceramide treatment could induce mitochondrial apoptosis by directly activating MOMP (mitochondria outer membrane permeabilisation) and PP2A. MOMP could then disrupt the outer mitochondrial membrane (OMM) and mediate the subsequent release of death-promoting proteins, such as cytochrome C, whereas PP2A could dephosphorylate and inactivate the anti-apoptotic protein BCL-2 and partially mediate Akt dephosphorylation. However, when the CERAMIDE node was activated alone, the mitochondrial apoptotic pathway was inhibited by activated AKT. AKT activated anti-apoptotic BCL-2 family proteins, such as BCL-2 and BCL-xL, by phosphorylating and inhibiting pro-apoptotic BCL-2 family proteins, such as BAD, BIM and BAX. Through the activation of mTOR, AKT can also inhibit PP2A and prevent the dephosphorylation and inactivation of BCL-2^48^. Accordingly, a combination of BCL2 inhibitor ABT263 and C2-ceramide also had synergistic effect on cell viability (Fig. 5d). However, individual inhibition of the AKT node failed to activate mitochondrial apoptosis due to the absence of pro-apoptotic stress, such as the activation of CERAMIDE node. We found MOMP could only be fully activated when the CERAMIDE node was fixed in the ON state while the AKT node remained OFF, leading to the activation of downstream CYTC, CASP9 and CASP3, and consequently, cell apoptosis. To further validate this mechanism, we detected apoptosis using AnnexinV-propidium iodide dual staining. In accordance with our model, combined treatment of MK2206 and C2-ceramide showed strong synergistic apoptotic effects on HT29 cells (Fig. 5e). C2-ceramide or MK2206 alone induced approximately 20% cell apoptosis, whereas their combination significantly increased the apoptosis level to 40–60% (Fig. 5e). These results agree with those obtained by the combination of siRNA against AKT1, 2 and 3 and C2-ceramide (Fig. 5f and Supplementary Fig. S6b). We consistently observed the cleavage of caspase 3, 8 and 9 and PARP following combined treatment with MK2206 and C2-ceramide (Fig. 5g). The occurrence of apoptosis stemmed from a key mitochondrial event, namely cytochrome C release, which was not induced by individual treatments compared with the control group but was significantly increased after the combination of MK2206 and C2-ceramide (Fig. 5h).

To further explore the clinical potential of our combination strategy, we tested combinations of marketed chemotherapeutic drugs. Epidermal growth factor receptor (EGFR) is one of the upstream tyrosine kinases of the PI3K/AKT pathway. Dosing HT-29 cells with C2-ceramide together with lapatinib or gefitinib, two clinically used EGFR inhibitors, exerted synergistic effects on cell viability (Supplementary Fig. S6c and S6d). In addition, PI3K/AKT inhibitors and FTY720 (fingolimod), which has previously been shown to inhibit and degrade SPHK1^49^, and conversely increase ceramide levels^50^, also had combinatory effects, although to a lesser extent than C2-ceramide (Supplementary Fig. S6e and S6f). In agreement with our previous findings that FTY720 stimulates endogenous ceramide accumulation by modulating sphingolipid metabolism^50^, FTY720 treatment elevated ceramide levels in HT-29 cells (Supplementary Fig. S6g).

Taken together, experimental validations of the effective drug combinations and related mechanisms in colon cancer cells further supported the rationality of our model. Most importantly, the combination strategy provides a mechanism-based rational therapeutic approach for CAC, as well as for other inflammation-associated cancers.

## Discussion

In this article, we presented a reconstruction of the CAC network and its implementation as a discrete dynamic Boolean model. The rationality of the CAC network model was justified by comparing the simulation results with existing observations, as well as novel experimental validations. Comprehensive analysis of the dynamic properties of the CAC network was also performed to unravel the missing link between chronic inflammation and cancer development and to identify potential therapeutic targets.

Through manipulation of the nodes in the microenvironment component of the CAC network model, we suggests that dendritic cells play critical roles in forming the pro-proliferative inflammatory microenvironment. The transient activation of DCs initiates a controlled inflammatory reaction and eventually leads to an immune suppressive microenvironment, whereas the sustained activation of DCs leads to a dysregulated inflammatory microenvironment, which strongly supports the proliferation and survival of IECs. In addition, we thoroughly characterized the dynamic properties of CAC network under the pro-tumor inflammatory microenvironment and identified a core regulatory network that governed the cell outcome. According to the core regulatory network, P53 inactivation was found to be critical for malignant transformation of epithelial cells under the pro-tumor inflammatory microenvironment. The result was supported by current findings of a dysregulated P53 pathway during CAC development and partly explained that in the ‘two-hit’ model, a somatic mutation is necessary for tumour initiation in a pro-tumour inflammatory microenvironment^39^.

**Figure 6:**
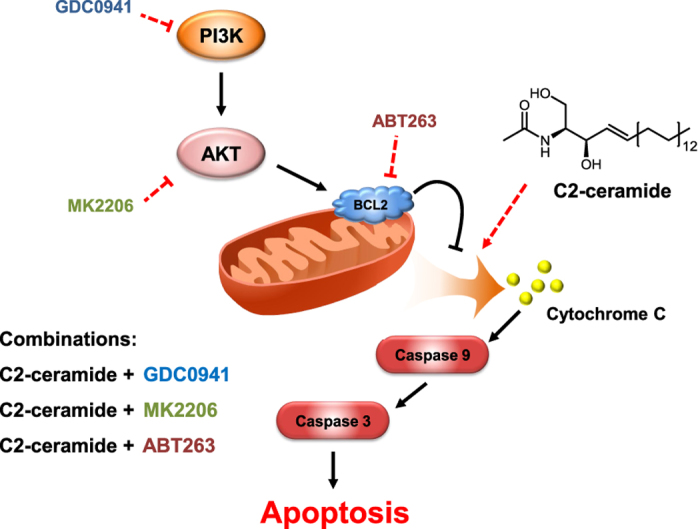
Proposed model for the synergistic effect between ceramide and PI3K/AKT pathway inhibitors in inducing apoptotic cell death of cancer cells. Ceramide directly or indirectly targets mitochondria, leading to the release of apoptotic proteins, such as cytochrome C, and activates caspases. However, if the PI3K/AKT pathway is activated by an inflammatory stimulus or other growth factors, it can preserve the integrity of the mitochondrial outer membrane by activating the anti-apoptotic BCL-2 family of proteins, such as BCL-2 or BCL-xL, by inhibiting PP2A or BAD. Therefore, ceramide and PI3K/AKT pathway inhibitors exert a synergistic cytotoxic effect on cancer cells. Note: the arrows in this simplified scheme are not intended to indicate direct physiological interactions.

Subsequent systematic perturbation analysis indicated that various dysregulations could facilitate the malignant transformation of preneoplastic IECs in an inflammatory microenvironment, including the hyperactivation of the NF-κB, PI3K/AKT or the COX2 pathways. Many perturbations were also predicted to suppress cell survival and proliferation of IECs in an inflammatory microenvironment, such as the inhibition of the ERK/MAPK pathway or the SPHK1/S1P pathway. These perturbations can be used as an early intervention method to prevent CAC development. However, the CAC network in a P53 inactive state, which mimicked malignant transformed IECs, was more resistant to perturbations. Double perturbation studies on the P53-inactivated CAC network suggested that combinatorial intervention methods through multi-targeted drugs or drug combinations could be more effective at treating later-stage CAC patients. Most importantly, we discovered and validated some novel combinatorial therapeutic approaches. We found that simultaneously inhibiting PI3K/AKT signalling and adding C2-ceramide, a pro-apoptotic sphingolipid signalling molecule, had a synergistic cytotoxic effect on colon cancer cells under an inflammatory stimulus. We explored the underlying mechanism of this synergistic effect by combining biochemical experiments and network simulations. We demonstrated that this effect was primarily attributed to regulation of the mitochondrial apoptotic pathway. As a pro-apoptotic signalling molecule, ceramide can directly or indirectly target mitochondria and lead to the release of apoptotic proteins, such as cytochrome C, and initiate the apoptosis machinery^51^,^52^. However, in many tumour cells, the PI3K/AKT pathway is highly active, which could in turn adversely affect the integrity of the mitochondria outer membrane by indirectly activating anti-apoptotic BCL-2 family proteins, such as BCL-2 or BCL-xL^53^. Our simulation results and previous experimental findings also suggest that inflammatory signalling can activate the AKT pathway to enhance survival of tumour cells. Therefore, only when PI3K/AKT signalling is blocked can the apoptosis machinery effectively be activated by a pro-apoptotic stimulus, such as ceramide (Fig. 6). Our results may also explain previous findings in which D, L-threo-1-phenyl-2-decanoylamino-3-morpholino-1-propanol (PDMP), a modulator of ceramide metabolism that elevates endogenous ceramide levels, could sensitise leukemic cells to the treatment of ABT263, an inhibitor of anti-apoptotic BCL2-like proteins^54^.

We are aware that this model is unable to capture the full complexity of CAC development. Some unknown factors may be present in CAC development, and merely incorporating outcomes, such as proliferation and apoptosis, is insufficient to assess tumour development. However, the basic property of tumour cells is uncontrolled proliferation, and inducing growth arrest or apoptosis is the most effective method to prevent cancer development. Therefore, our theoretical model can provide valuable information to guide further experimental studies, and this model can be easily refined and expanded with the availability of additional information.

In conclusion, the dynamic modelling of the CAC development process can lead to a better mechanistic understanding of CAC and other inflammation-associated cancers because CAC serves as a paradigm for inflammation-associated cancer development. Our model and experimental findings will also be helpful in identifying novel therapeutic targets and the design of combinatorial therapeutic approaches to achieve early prevention and treatment of CAC.

## Materials and Methods

### Construction of the CAC network

A hierarchical method was used to construct the CAC network. First, signalling components that are critical participants in IBD and colorectal cancer, as indicated by a literature search, were collected. These components were used as seed nodes to build the initial network model. Then, the initial network was expanded using the GeneGo database (http://www.genego.com/). The regulatory relationships were then verified manually, and the interactions that were not specifically mentioned to be relevant to colitis or colon cancer were removed. Further, a similar approach, such as the one Zhang *et al.*^13^,^55^ used in constructing T-LGL network model, was adopted to remove the redundant and indirect interactions between nodes.

### Boolean dynamic modelling

In the Boolean model, each node has only two discrete states: ON and OFF (1 and 0). The regulatory relationships between upstream nodes (regulators) and downstream nodes (targets) are expressed by the logical operators AND, OR and NOT^56^. The future state of each node can be determined by the current states of the nodes and the Boolean transfer function 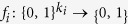, where *k*_*i*_ is the number of regulators of node *i*. In Boolean models, the time variable is discrete and usually designated as steps. To propagate the discrete states of a Boolean model, different node-updating strategies have been proposed, such as synchronous and asynchronous update methods^21^. In this study, we mainly used a general asynchronous (GA) method^57^, in which a randomly selected node is updated at each time step. To evaluate the general behaviour trends for all nodes in the network model, multiple replicate simulations were performed from the same initial conditions but with random update orders. These trends can be reflected by the activation frequency for each node, which was calculated by dividing the number of simulations where the node is ON by the total replicate number. The GA method has been widely used in modelling signal transduction networks and has been suggested to be the most informative and computationally efficient method among asynchronous updating strategies^21^,^56^. We also compared the simulation results using synchronous method, GA method and another asynchronous updating method—random order asynchronous (ROA) method^58^.

### Network reduction

Considering that the size of the state space of a Boolean model is exponentially dependent on the node number (a Boolean network with *n* nodes has 2^*n*^ states) and attractor identification is a strong NP-complete problem, tracking all of the attractors within a relatively small network is computationally demanding. Therefore, we used a network reduction method proposed by Saadatpour *et al.*^21^ to reduce the node number while maintaining the long-term behaviour of the dynamic model. First, the nodes that stabilise in an attracting state (ON or OFF) during the entire simulation are eliminated. The attracting states of these nodes are only determined by the states of the input nodes and can be readily identified by inspecting the Boolean functions. Second, the simple mediator nodes, with both in-degrees and out-degrees equal to one, are iteratively removed, and their input and output nodes are connected directly. The dangling nodes (nodes with zero out-degrees) are also removed. This method can effectively reduce the network size and maintain the fixed point, as well as the complex attractor of Boolean models, using either synchronous or GA methods.

### Attractor identification

When updating a Boolean model, the state of the whole system at a certain time step is defined by the collection of the states of all the nodes at that step. As the Boolean model evolves over time, all the possible states of the system constitute the state space, which can be represented by a state transition graph whose nodes are the states of the system and the edges are allowed transitions among states. Attractors are special states in the state transition graph that the system will eventually settle down to and will not transit to other states. An attractor can either be a fixed point, in which the state of the system does not change, or a complex attractor, in which the states traverse regularly or irregularly over a series of states^56^.

To identify the attractors of the reduced CAC network model, we firstly constructed the state transition graph by updating the model starting from all possible initial states. Subsequently, the strongly connected components (SCCs) of the state transition graph were identified. SCCs are subgraphs of a directed graph in which every node is reachable from every other node. Complex attractors of a Boolean model are the SCCs with empty out-component of the state transition graph^59^. Fixed point attractors can also be identified by this approach because a fixed point can be regarded as an SCC that contains only one state and is strongly connected to itself. The states in the state transition graph that can reach a certain attractor were marked as the basin states of that attractor.

### Coding

We implemented the functions used in this study into an open source Python software package named SimpleBool, which can be downloaded at https://github.com/SimpleBool/SimpleBool. SimpleBool directly reads model and parameter input files to perform dynamic simulations, attractor identification and *in silico* perturbation studies. SimpleBool can run in a stand-alone mode, and therefore, coding experience is not required. Readers can refer to the website for further guidance on how to use the software.

### Antibody and compounds

Antibodies against STAT3, p-STAT3 (Y705), P65, p-P65 (S536), AKT, p-AKT (S473), PARP, cleaved-caspase 3, cleaved-caspase 8 and cleaved-caspase 9 were obtained from Cell Signalling Technology. Anti-GAPDH antibody was from Epitomics. MK2206, GDC0941 and ABT263 were purchased from Selleckchem.

### Cell line, cell culture and *in vitro* experiments

HT29 cells were obtained from the American Type Culture Collection (Rockville, USA). Cell line identity is routinely monitored by short tandem repeat (STR) analysis. Cell lines were grown at 37 °C in a 5% CO_2_ incubator. The cell medium was McCoy’s 5a (Gibco) supplemented with 10% FBS; 50 U/ml penicillin, 50 U/ml streptomycin.50 ng/ml IL6 (PeproTech) and 10 ng/ml TNF-α (PeproTech) were added to the medium to model an inflammatory microenvironment. Cell viability was measured using the Cell Proliferation Reagent sulforhodamine B (SRB, sigma). For siRNA transfection, cells were plated at 3 × 10^5^ cells/ml in OPTI-MEM serum-free medium and transfected with a specific siRNA duplex using Lipofectamine® RNAiMAX Reagent Agent (Life Technologies) according to the manufacturer’s instructions. Synergy between combination treatments was determined by the combination index using CompuSyn software, available online.

### Flow cytometry

Cells were plated and treated the following day with the indicated agents. Cells were detached using trypsin-EDTA, resuspended in growth medium and counted. To detect cell apoptosis using Annexin V/propidium iodide staining, 1 × 10^6^ cells were washed with cold PBS, resuspended in 100 μl of binding buffer, and then, propidium iodide and FITC-labelled antibody against annexin V were added according to the manufacturer’s protocol (Vazyme Biotech). FlowCellect Cytochrome c Reagents (Millipore) were obtained for analysis of cytochrome C release from mitochondria. Briefly, 1 × 10^5^ cells were washed with PBS, and then, a permeabilsation buffer working solution and a fixation buffer working solution were added sequentially to achieve selective permeabilisation of mitochondria while leaving the mitochondrial membrane intact. Finally, 10 μl of either the anti-IgG1-FITC isotype control or the anti-cytochrome c-FITC antibody was added to each sample according to the manufacturer’s protocol. At least 3 × 10^3^ cells per sample were analysed with a FACScan flow cytometer (Becton Dickinson).

### Statistical analysis

Data are representative of three independent experiments (mean ± s.e.m.). A two-tailed Student’s t-test was used for statistical comparisons between groups, and P-values ≤ 0.05 were considered statistically significant.

## Additional Information

**How to cite this article**: Lu, J. *et al.* Network modelling reveals the mechanism underlying colitis-associated colon cancer and identifies novel combinatorial anti-cancer targets. *Sci. Rep.* **5**, 14739; doi: 10.1038/srep14739 (2015).

## Supplementary Material

Supplementary Information

## Figures and Tables

**Table 1:** Activation frequencies of Proliferation, Apoptosis and inflammatory cytokines for different combinatorial activations of immune cells.

	DC	MAC	TREG	CTL	TH1	TH2	Proliferation	Apoptosis	TNFA	IL6	TGFB	IFNG	IL10	IL12	IL4
**1**	**•**						0.66	0	0.06	1	0	0.06	0.94	1	1
**2**		**•**					0.5	0	1	1	0	0.58	0.42	1	1
**3**			**•**				0.21	0.55	0	0.21	1	0	1	0	0
**4**				**•**			0.5	0	1	1	0	1	0	1	1
**5**					**•**		0.5	0	1	1	0	1	0	1	1
**6**						**•**	0.22	0.54	0	0.22	0.78	0	1	0	1
**7**	**•**		**•**				0.59	0	0	1	1	0	1	1	1
**8**	**•**			**•**			0.5	0	1	1	0	1	0	1	1
**9**	**•**		**•**	**•**			0.43	0.57	0	1	1	1	1	1	1
**10**	**•**	**•**	**•**	**•**			0.5	0	1	1	1	1	1	1	1
**11**			**•**	**•**			0.17	0.83	0	0.17	1	1	1	0	0
**12**	**•**			**•**		•****	0.58	0.42	0	1	0	1	1	1	1
**13**	**•**	**•**		**•**		**•**	0.49	0	1	1	0	1	1	1	1
**14**				**•**		**•**	0.17	0.83	0	0.17	0.83	1	1	0	1

**•** indicates that the immune cell node in the header of the corresponding column was fixed in the ON state.

**Table 2:** Attractors of the reduced CAC network shown in Fig. 3b.

ID	Type	Length	Basin size	Exclusive basin size	Phenotype
1	Complex attractor	48	75%	12.5%	Limited proliferation
2	Limited cycle	6	75%	2.05%	Proliferation
3	Limited cycle	6	70%	0.03%	Resting

The attractors can have shared basins since the general asynchronous updating method was used. Therefore, both the total basin size and the exclusive basin size were calculated for each attractor.

**Table 3:** Double perturbations that may sensitise IECs to pro-apoptotic signals.

Nodes	BAX(+)	TBID(+)	FAS(+)	CYTC(+)	CASP8(+)	CASP9(+)	CERAMIDE(+)
AKT(−)	0.06/0.4	0.06/0.41	0.06/0.68	0.05/0.34	0.06/0.69	0.06/0.47	0/1
PI3K(−)	0.06/0.4	0.06/0.41	0.07/0.67	0.06/0.34	0.07/0.68	0.06/0.47	0/1
PTEN(+)	0.06/0.4	0.06/0.41	0.06/0.68	0.05/0.35	0.07/0.68	0.06/0.47	0/1
PP2A(+)	0/1	0/1	0.06/0.94	0.06/0.34	0/1	006/0.47	0/1
BCL2(−)	0/1	0/1	NA	NA	0/1	NA	0/1
RAS(−)	0/0.41	0/0.41	0/0.84	0/0.35	0/0.69	0/0.48	0.06/0.94
IL6(−)	0/1	0/1	0/1	0/1	0/1	0/1	0/1
GP130(−)	0.06/0.94	0.06/0.91	0.06/0.94	0.05/0.95	0.05/0.95	0.06/0.94	0.06/0.94
IKK(−)	0.37/0.45	0.33/0.46	0.12/0.72	0.4/0.38	0.12/0.72	0.25/0.51	0/1
IKB(+)	0.33/0.46	0.33/0.46	0.1/0.73	0.38/0.38	0.1/0.73	0.22/0.52	0/1
NFKB(−)	0.33/0.46	0.33/0.46	0.1/0.73	0.37/0.39	0.1/0.73	0.22/0.51	0/1
IAP(−)	NA	NA	NA	0/1	0/1	0/1	NA

(+) indicates fixing the node in the ON state (activation), (−) indicates fixing the node in the OFF state (inhibition). The results are shown as the activation frequency of Proliferation/the activation frequency of Apoptosis, and NA indicates no additional effect on Apoptosis and Proliferation compared with single perturbation.
