# Supplementary material for: Network modelling reveals the mechanism underlying colitis-associated colon cancer and identifies novel combinatorial anti-cancer targets
Source: Sci Rep. 2015 Oct 8;5:14739. doi: 10.1038/srep14739 (PMC4597205; doi:10.1038/srep14739)
Supplement: Supplementary Information [file srep14739-s1.pdf]

Supplementary Information for

Network modelling reveals the mechanism underlying colitis-associated colon cancer and identifies novel combinatorial anti-cancer targets

Junyan Lu[†¶], Hanlin Zeng[†¶], Zhongjie Liang[#¶], Limin Chen[†], Liyi Zhang[†], Hao Zhang[†], Hong Liu[†], Hualiang Jiang[†], Bairong Shen[#], Ming Huang[*], Meiyu Geng[*] Sarah Spiegel[$] and Cheng Luo[†#*]

[†]State Key Laboratory of Drug Research, Shanghai Institute of Materia Medica, Chinese Academy of Sciences. [#]Soochow University, Center for Systems Biology, Jiangsu, China. [$]Department of Biochemistry and Molecular Biology, Virginia Commonwealth University School of Medicine, Richmond, VA 23298,USA.

[¶] These authors contributed equally.

[*]To whom correspondence should be addressed. Email: mhuang@simm.ac.cn (Ming Huang) or mygeng@simm.ac.cn (Meiyu Geng） or cluo@simm.ac.cn (Cheng Luo). Tel: +86-21-50806600; Fax: +86-21-50807188

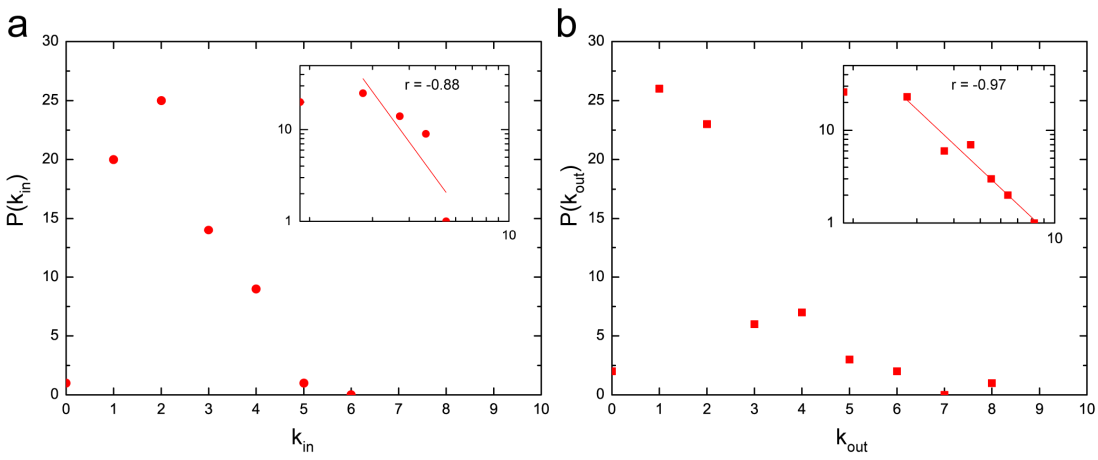

Supplementary Figure S1. Node degree distributions for the CAC network. (a) In-degree distribution *P(k_{in})* and out-degree distribution *P(k_{out})* for the CAC network. It is worth noting that, as we removed dangling nodes (nodes with zero out-degree and only one in-degree) to simplify the network topology, the number of nodes with in-degree of one is decreased and deviate from the power-law distribution. However, this simplification should not impact the dynamic properties of the CAC network, with which we are more concerned. The insets suggest the degree distributions follow power-law distributions if the nodes of degree one are omitted.

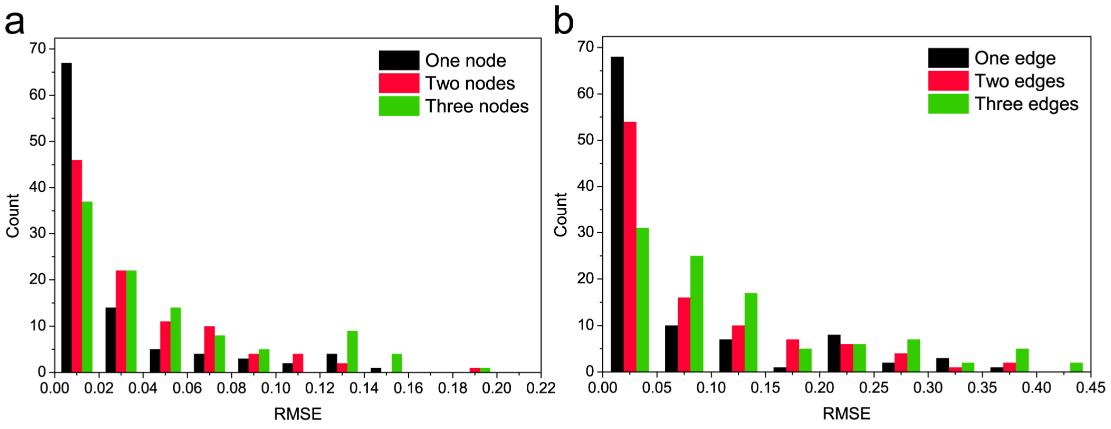

Supplementary Figure S2. Robustness test of the CAC network. (a) Perturbation of the initial node states. One hundred initial states were randomly generated and the states of one, two or three randomly chosen nodes were flipped. The averaged states of all the nodes were recorded, after 5,000 rounds of iterations using the general asynchronous updating methods from perturbed and unperturbed initial states. The root-mean-square error (RMSE) between the simulation results from unperturbed initial states and perturbed ones were calculated. According to the distribution of RMSE values, the majority of the RMSE values are small (between 0.00 and 0.02), indicating the network model is robust to small amounts of noise in the initial network states. (b) Perturbation of deleting interaction (edge). One hundred initial states were randomly generated and one, two or three randomly chosen edges were deleted. In a similar manner, the RMSE values between the simulations results of the unperturbed networks and perturbed ones were calculated. The results also indicate the network model is robust to small changes in the topology structure.

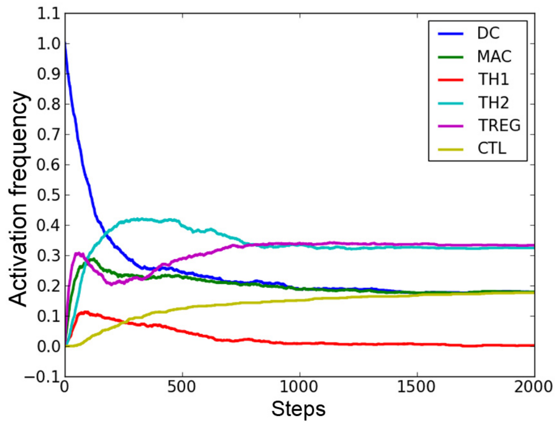

Supplementary Figure S3. The fluctuation of the activation frequencies of the immune cell nodes when the initial state of DC was set in ON state (transient activation).

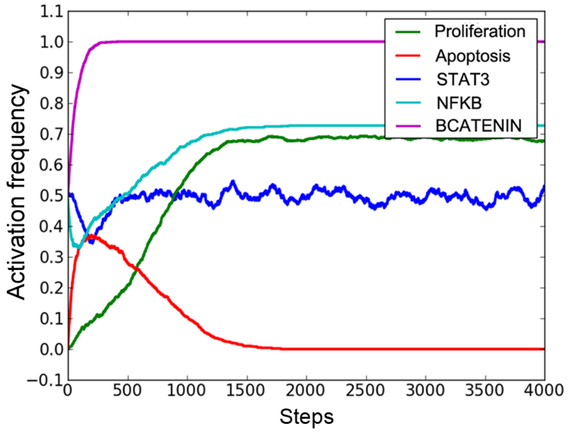

Supplementary Figure S4. The activation frequencies of Proliferation, Apoptosis, STAT3, NFKB and BCATENIN under the pro-tumor inflammatory microenvironment (DC in ON state) while APC was kept in OFF state, mimicking the APC inactive mutation observed in colorectal cancers.

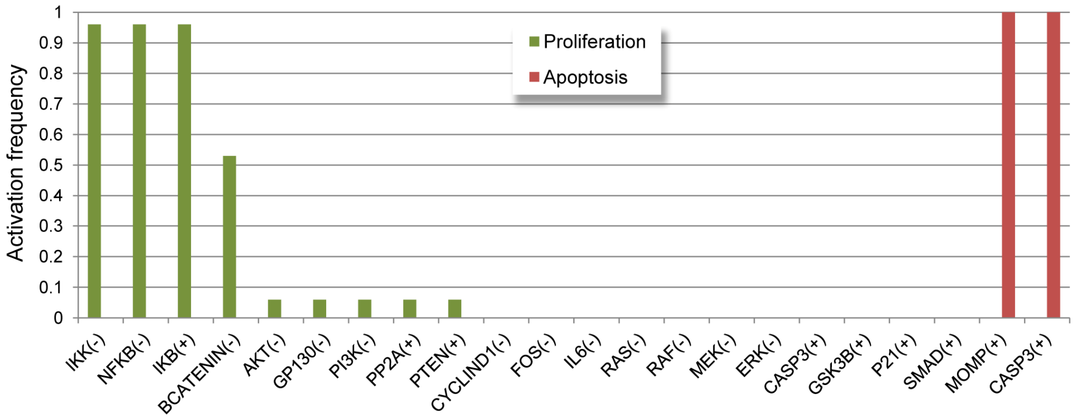

Supplementary Figure S5. Node perturbations on the CAC network model in P53 inactive state.

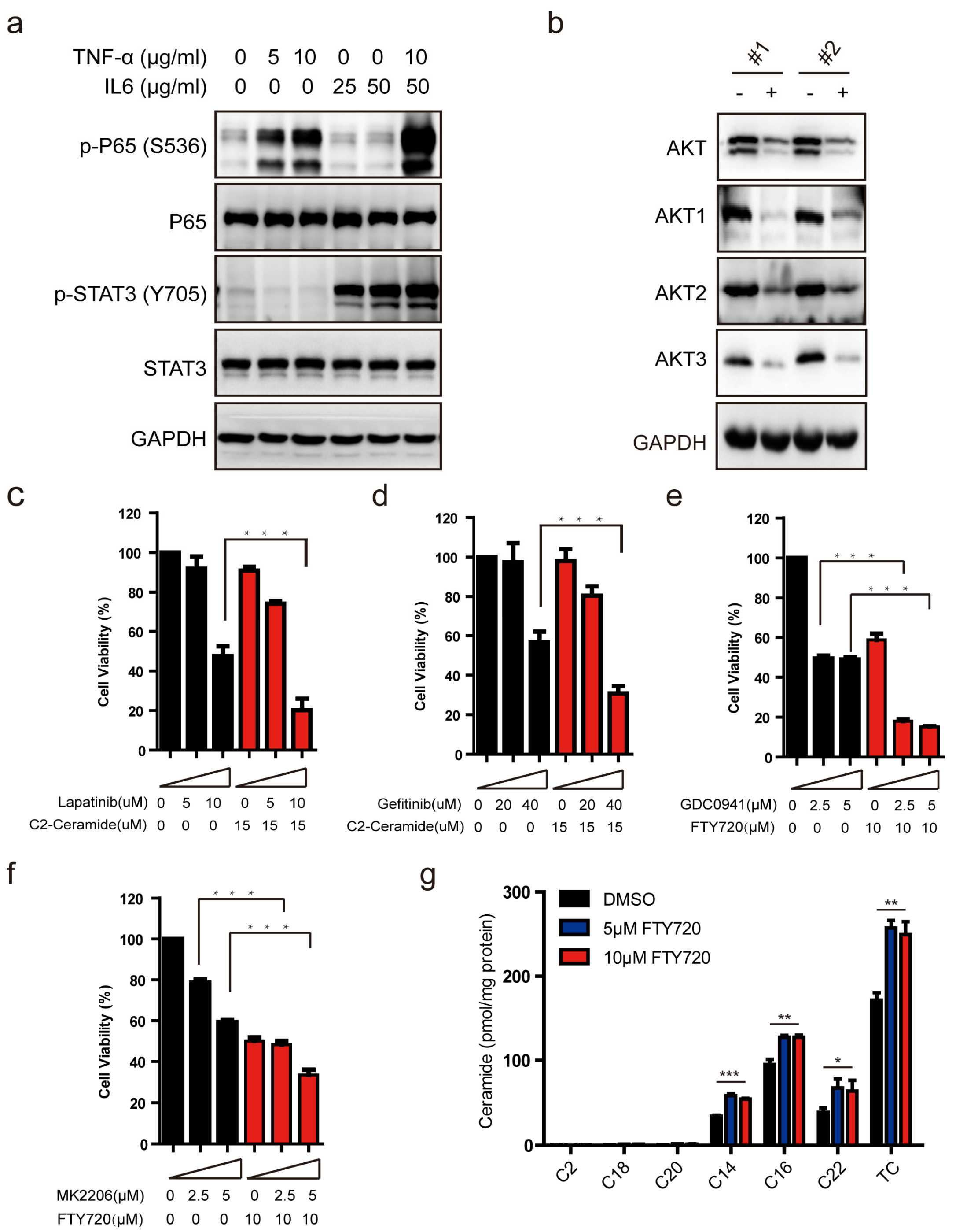

Supplementary Figure S6. (a) Immunoblots of lysates of HT29 cells after treatment with inflammatory cytokine IL6 and TNF-α for 30min. (b) Immunoblots of lysates from cells in which AKT1,2,3 were depleted by siRNA. (c-f) Cell viabilities detected at 48 h after combinatorial treatments of Lapatinib and C2-ceramide (c), Gefitinib and C2-ceramide (d), GDC0941 and FTY720 (e) and FTY720 and MK2206 (f). (g) Ceramide levels in HT29 cells determined by HPLC-ESI-MS/MS assay after FTY720 treatment for 6 h. TC means total ceramide (C2-, C14-, C16-, C18-, C20-, and C22-ceramide). The intracellular concentrations of C2-, C18- and C20- ceramides were very low compared with that of C14-, C16- and C22-ceramides.

Supplementary Table S1. The full names of components in the CAC network corresponding to the abbreviated node labels used.

Node name	Gene symbol	Full name
AKT	AKT1/AKT2	v-akt murine thymoma viral oncogene homolog 1 / v-akt murine thymoma viral oncogene homolog 2
APC	APC	adenomatous polyposis coli
ASK1	MAP3K5	apoptosis signal-regulating kinase 1 (also known as mitogen-activated protein kinase kinase kinase 5)
ATM	ATM	ataxia telangiectasia mutated
BAX	BAX	BCL2-associated X protein
BCATENIN	CTNNB1	beta-catenin
BCL2	BCL2	B-cell CLL/lymphoma 2
CASP3	CASP3	caspase 3, apoptosis-related cysteine peptidase
CASP8	CASP8	caspase 8, apoptosis-related cysteine peptidase
CASP9	CASP9	caspase 9, apoptosis-related cysteine peptidase
CCL2	CCL2	chemokine (C-C motif) ligand 2
CERAMIDE	N/A	ceramide
CFLIP	CFLAR	CASP8 and FADD-like apoptosis regulator
COX2	PTGS2	prostaglandin-endoperoxide synthase 2 (prostaglandin G/H synthase and cyclooxygenase)
CTL	N/A	CD8+ cytotoxic lymphocyte
CYCLIND1	CCND1	cyclin D1
CYTC	CYCS	cytochrome c, somatic
DC	N/A	dendritic cells
EP2	PTGER2	prostaglandin E receptor 2 (subtype EP2), 53kDa
ERK	MAPK1/MAPK3	mitogen-activated protein kinase 1/ mitogen-activated protein kinase 3 (also known as ERK1 and ERK2)
FADD	FADD	Fas (TNFRSF6)-associated via death domain
FAS	FAS	Fas (TNF receptor superfamily, member 6)
FOS	FOS	FBJ murine osteosarcoma viral oncogene homolog
GP130	IL6ST	interleukin 6 signal transducer (gp130, oncostatin M receptor)
GSK3B	GSK3B	glycogen synthase kinase 3 beta
IAP	XIAP/BIRC3/BIRC2	X-linked inhibitor of apoptosis / baculoviral IAP repeat containing 3 / baculoviral IAP repeat containing 2
IFNG	IFNG	interferon, gamma
IKB	NFKBIA/NFKBIB	nuclear factor of kappa light polypeptide gene enhancer in B-cells inhibitor, alpha/beta
IKK	CHUK/IKBKB	conserved helix–loop–helix ubiquitous kinase / inhibitor of kappa light polypeptide gene enhancer in B-cells, kinase beta (also known as IKK-alpha and IKK-beta)
IL10	IL10	interleukin 10

Node name	Gene symbol	Full name
IL12	IL12A	interleukin 12A
IL4	IL4	interleukin 4
IL6	IL6	interleukin 6 (interferon, beta 2)
JAK	JAK2	Janus kinase 2
JNK	MAPK8	mitogen-activated protein kinase 8
JUN	JUN	jun proto-oncogene
MAC	N/A	macrophages
MDM2	MDM2	Mdm2, p53 E3 ubiquitin protein ligase homolog (mouse)
MEK	MAP2K1/MAP2K2	mitogen-activated protein kinase kinase 1 / mitogen-activated protein kinase kinase 2 (also known as MEK1 and MEK2)
MEKK1	MAP3K1	mitogen-activated protein kinase kinase kinase 1, E3 ubiquitin protein ligase
MOMP	N/A	mitochondrial outer membrane permiabilization
NFKB	NFKB1/NFKB2/RELA	nuclear factor of kappa light polypeptide gene enhancer in B-cells 1 (p105) / nuclear factor of kappa light polypeptide gene enhancer in B-cells 2 (p49/p100) / v-rel reticuloendotheliosis viral oncogene homolog A, nuclear factor of kappa light polypeptide gene enhancer in B-cells 3, p65 (avian)
P21	CDKN1A	cyclin-dependent kinase inhibitor 1A (p21, Cip1)
P53	TP53	tumor protein p53
PGE2	N/A	prostaglandin E2
PI3K	PIK3CA/PIK3CB	phosphoinositide-3-kinase, catalytic, alpha polypeptide / phosphoinositide-3-kinase, catalytic, beta polypeptide
PP2A	PPP2CA/PPP2CB	protein phosphatase 2, catalytic subunit, alpha isoform / protein phosphatase 2, catalytic subunit, beta isozyme
PTEN	PTEN	phosphatase and tensin homolog
RAF	RAF1	v-raf-1 murine leukemia viral oncogene homolog 1
RAS	KRAS/HRAS	v-Ki-ras2 Kirsten rat sarcoma viral oncogene homolog / v-Ha-ras Harvey rat sarcoma viral oncogene homolog
ROS	N/A	reactive oxygen species
S1P	N/A	Sphingosine-1-phosphate
SMAC	DIABLO	diablo, IAP-binding mitochondrial protein
SMAD	SMAD2/SMAD3/SMAD4	SMAD family member 2/3/4
SMAD7	SMAD7	SMAD family member 7
SMASE	SMPD1/SMPD2/SMPD4	sphingomyelin phosphodiesterase 1, acid lysosomal / sphingomyelin phosphodiesterase 2, neutral membrane (neutral sphingomyelinase) / sphingomyelin phosphodiesterase 4, neutral membrane (neutral sphingomyelinase-3)
SOCS	SOCS1	suppressor of cytokine signaling 1

Node name	Gene symbol	Full name
SOD	SOD1/SOD2	superoxide dismutase 1, soluble / superoxide dismutase 2, mitochondrial (also known as Cu/ZN-SOD and Mn-SOD)
SPHK1	SPHK1	sphingosine kinase 1
STAT3	STAT3	signal transducer and activator of transcription 3 (acute-phase response factor)
TBID	BID	BH3 interacting domain death agonist
TGFB	TGFB1/TGFB2	transforming growth factor, beta 1 / transforming growth factor, beta 2
TGFR	TGFBR2	transforming growth factor, beta receptor II (70/80kDa)
TH1	N/A	type 1 T helper cells
TH2	N/A	type 2 T helper cells
TNFA	TNF	tumor necrosis factor
TNFR	TNFRSF1A	tumor necrosis factor receptor superfamily, member 1A
TREG	N/A	regulatory T cells

Supplementary Table S2. Topology properties of the CAC network and random networks with the same node and edge number.

Network property	CAC network	Random networks
Nodes	70	70
Edges	153	153
Average node connectivity	3.15	3.20±0.1
Clustering coefficient	0.10	0.06±0.02
Shortest path length	3.37	2.98±0.05

The average values and standard deviations of the network properties were calculated from a ensemble of 1,000 random networks.

Supplementary Table S3. Boolean logical rules that govern the state of the nodes in CAC network.

ATM* = ROS
ASK1* = ROS and not P21
AKT* = PI3K and not (PP2A or CASP3)
BAX* = ((TBID or P53) and PP2A) and not AKT
BCATENIN* = not (GSK3B and APC)
BCL2* = (STAT3 or NFKB) and not (P53 or PP2A)
CASP3* = (CASP8 or CASP9) and not IAP
CASP8* = FADD and not (CFLIP or P21)
CASP9* = CYTC and not (IAP or P21)
CERAMIDE* = SMASE and not SPHK1
CFLIP* = NFKB
COX2* = S1P and TNFR
CYCLIND1* = (BCATENIN or STAT3 or JUN) and not GSK3B
CYTC* = MOMP
EP2* = PGE2
ERK* = MEK
FAS* = CTL
FADD* = TNFR or FAS
FOS* = ERK
GP130* = IL6
GSK3B* = not (EP2 or AKT)
IAP* = (NFKB or STAT3) and not SMAC
IKB* = not IKK
IKK* = (AKT or (S1P and TNFR))
JAK* = GP130 and not SOCS
JNK* = ASK1 or MEKK1
JUN* = ((BCATENIN or ERK) and JNK) and not GSK3B
MDM2* = (P53 and AKT) and not (GSK3B or ATM)
MEK* = RAF or ROS
MEKK1* = CERAMIDE or TGFR or TNFR
MOMP* = (BAX or TBID or CERAMIDE) and not BCL2
NFKB* = not IKB
P21* = (P53 or SMAD) and not (GSK3B or CASP3)
P53* = (PTEN or JNK or ATM) and not MDM2
PGE2* = COX2
PI3K* = (EP2 or RAS) and not PTEN
PP2A* = CERAMIDE and not AKT
PTEN* = P53 and not (NFKB or JUN)
RAF* = CERAMIDE or RAS
RAS* = EP2 or GP130
ROS* = TNFR and not SOD
SOD* = NFKB or STAT3

S1P* = SPHK1
SMAC* = MOMP
SMAD* = TGFR and not JUN
SMAD7* = SMAD or NFKB
SMASE* = P53 or FADD
SPHK1* = ERK or TNFR
STAT3* = JAK
SOCS* = STAT3
TBID* = CASP8 and not BCL2
TGFR* = TGFB and not SMAD7
TNFR* = TNFA
TREG* = (IL10 or DC) and not IL6
TNFA* = MAC
TH2* = IL4 and not (IFNG or TGFB)
TH1* = (IL12 or IFNG) and not (IL10 or TGFB or IL4)
TGFB* = TREG
MAC* = (IFNG or CCL2) and not IL10
IL6* = MAC or DC or NFKB
IL4* = DC or TH2
IL12* = DC or MAC
IL10* = TREG or TH2
IFNG* = TH1 or CTL
CTL* = IFNG and not TGFB
DC* = (CCL2 or TNFA) and not IL10
CCL2* = NFKB
Proliferation* = (FOS and CYCLIND1) and not (P21 or CASP3)
Apoptosis* = CASP3

"Node*" denotes the next state of the same node on the left-hand side of the equation, which is determined by the states of its regulators according to the function on the right-hand side of the equation.

Supplementary Table S4. Attractors of the CAC network under the non-inflammatory microenvironment and normal inflammatory response.

Conditions	Attractors					
	ID	Type	Length	[2]Basin size	[2]Exclusive basin size	Phenotype
**Non-inflammatory microenvironment**	1	Fixed point	1	75.0%	6.25%	Resting
	2	Complex attractor	192	93.8%	25.0%	[1]Limited apoptosis
**Normal inflammatory response**	1	Fixed point	1	13.6%	0%	Resting
	2	Fixed point	1	34.4%	1.12%	Apoptosis
	3	Complex attractor	96	16.7%	11.1%	Proliferation
	4	Complex attractor	6	56.0%	10.5%	Proliferation
	5	Complex attractor	1345	19.5%	2.94%	Apoptosis

[1]Under the non-inflammatory microenvironment, the Apoptosis node oscillated between ON and OFF in the complex attractor, and therefore this attractor represented a limited apoptosis phenotype.

[2]The attractors can have shared basins since the general asynchronous updating method was used. Therefore, both the total basin size and the exclusive basin size were calculated for each attractor.

Supplementary Table S5. Boolean logical rules governing the nodes' states in the 21-node sub-network presented in Figure 3b.

AKT* = not PTEN
BCATENIN* = not (P53 and GSK3B)
CTL* = IFNG and not IL10
CYCLIND1* = (BCATENIN or STAT3 or JUN) and not GSK3B
GSK3B* = not (TNFR or AKT)
IFNG* = CTL
IKK* = AKT or TNFR
IL10* = not IFNG
JAK* = not SOCS
JNK* = TNFR
JUN* = JNK and not GSK3B
MAC* = (IFNG or NFKB) and not IL10
MDM2* = (P53 and AKT) and not GSK3B
NFKB* = IKK
P21* = P53 and not GSK3B
P53* = (PTEN or JNK) and not MDM2
PTEN* = P53 and not (NFKB or JUN)
SOCS* = STAT3
STAT3* = JAK
TNFR* = MAC
Proliferation* = CYCLIND1 and not P21

"Node*" denotes the next state of the same node on the left-hand side of the equation, which is determined by the states of its regulators according to the function on the right-hand side of the equation.

Supplementary Table S6. Attractors of the reduced CAC network model shown in figure 3b, when synchronous updating method was used.

ID	Cycle length	Basin size	Phenotype
**1**	2	0.19%	
**2**	6	0.57%	
**3**	6	0.02%	
**4**	6	0.71%	
**5**	2	0.23%	
**6**	2	0.01%	
**7**	6	0.02%	
**8**	6	0.70%	
**9**	6	0.71%	
**10**	2	0.24%	**Resting**
**11**	6	0.57%	
**12**	6	0.02%	
**13**	2	0.23%	
**14**	6	0.70%	
**15**	2	0.009%	
**16**	2	0.19%	
**17**	2	0.007%	
**18**	2	0.24%	
		**Sum: 5.4%**	
**19**	4	3.1%	
**20**	12	17%	
**21**	4	5.8%	
**22**	12	9.3%	
**23**	12	9.3%	**Limited Proliferation**
**24**	4	3.1%	
**25**	4	5.8%	
**26**	12	17%	
		**Sum: 71.5%**	
**27**	2	5.8%	
**28**	6	17.3%	**Proliferation**
		**Sum: 23.1%**	

All the attractors are limited cycles when synchronous updating method was used.

Supplementary Table S7. Comparison of the simulation results using different updating methods.

Condition	Updating method	Nodes' activation frequency				
		Proliferation	Apoptosis	STAT3	NFKB	BCATENIN
Non-inflammatory microenvironment	GA	0	0.36	0	0	0
	ROA	0	0.33	0	0	0
	Sync	0	0.28	0	0	0
Normal inflammation response	GA	0.40	0.30	0.25	0.50	0.53
	ROA	0.43	0.30	0.27	0.55	0.54
	Sync	0.74	0.18	0.43	0.76	0.75
Pro-tumor microenvironment	GA	0.63	0	0.51	0.69	0.69
	ROA	0.67	0	0.50	0.71	0.71
	Sync	0.88	0	0.50	0.92	0.91
Pro-tumor microenvironment and P53 inactivation	GA	1	0	0.46	1	1
	ROA	1	0	0.49	1	1
	Sync	1	0	0.50	1	1

GA: General asynchronous; ROA: Random order asynchronous; Sync: Synchronous

When different updating methods were used, the changes of the major simulation results showed similar trends under various conditions: under the non-inflammatory microenvironment, the Proliferation node stabilised in the OFF state while the Apoptosis node had a small chance to be activated (around 30%); The normal inflammation response (transient activation of the DC node) increased the activation frequency of Proliferation while the activation of Apoptosis was not significantly inhibited; The constant activation of the DC node, which formed the pro-tumor microenvironment, further increased the activation of Proliferation and inhibited Apoptosis, but the activation frequency of Proliferation did not reach 100%. Keeping P53 node at OFF state under pro-tumor microenvironment led all the simulation trajectories fall into the attractor that represents proliferation state. The impact of different microenvironments on the activation probabilities of STAT3, NFKB and BCATENIN, which represents the previously identified three major players in CAC, also followed a similar trend.

Supplementary Table S8. Comparison between the single node perturbation effects with experimental studies.

Node	Perturbation	Effect	Reference	Experiment model
P53	-	Pro-proliferative	[1, 2]	Colon tumor tissues
MDM2	+	Pro-proliferative	[3]	Patient samples
PI3K	+	Pro-proliferative	[4, 5]	Several colon cancer cell lines
AKT	+	Pro-proliferative	[6]	Colo205 and tumor tissues
PTEN	-	Pro-proliferative	[7]	Colon tumor tissues
NFKB	+	Pro-proliferative	[8]	Colon tumor tissue
IKK	+	Pro-proliferative	[9]	Mouse model
IKB	-	Pro-proliferative	[8]	Colon tumor tissue
JUN	+	Pro-proliferative	[10]	HCT116
CYCLIND1	+	Pro-proliferative	[11]	Colon tumor tissues
GSK3B	-	Pro-proliferative	[12]	SW480 and AGS
PGE2	+	Pro-proliferative	[13, 14]	Cell lines and mouse model
EP2	+	Pro-proliferative	[15]	Mouse model
COX2	+	Pro-proliferative	[13]	Colon cancer cell lines
AKT	-	Anti-proliferative	[16]	Clinical trials
PI3K	-	Anti-proliferative	[17, 18]	Cell lines and xenograft model
PTEN	+	Anti-proliferative	[19]	Colon cancer cell lines
ATM	+	Anti-proliferative	-	-
P53	+	Anti-proliferative	[20]	HCT116
P21	+	Anti-proliferative	[21]	HT29
SMAD	+	Anti-proliferative	-	-
ROS	+	Anti-proliferative	-	-
GSK3B	+	Anti-proliferative	-	-
FOS	-	Anti-proliferative	-	-
CYCLIND1	-	Anti-proliferative	[22]	SW480
GP130	-	Pro-apoptosis	[23]	SW480
RAS	-	Pro-apoptosis	[24]	Mouse model
RAF	-	Pro-apoptosis	[25, 26]	Colon cancer cell lines
PP2A	+	Pro-apoptosis	-	-
MEK	-	Pro-apoptosis	[26, 27]	Cell lines and mouse model
ERK	-	Pro-apoptosis	[26]	Colon cancer cell lines
IL6	-	Pro-apoptosis	[28]	Mouse model
SPHK1	-	Pro-apoptosis	[29]	Mouse model
CERAMIDE	+	Pro-apoptosis	-	-
MOMP	+	Pro-apoptosis	[30]	HCT116
CASP3	+	Pro-apoptosis	[31, 32]	Colo205 and SNU-C4

The shaded lines represent the *in silico* perturbation effects that have not been reported by experimental studies. These experimental results were not used in the model construction, and therefore they can regarded as the external sources to validate the CAC network model.

References for Supplementary Table 8:

1. Hasegawa H, *et al.* p53 gene mutations in early colorectal carcinoma. De novo vs. adenoma-carcinoma sequence. *International journal of cancer Journal international du cancer* **64**, 47-51 (1995).
2. Iacopetta B. TP53 mutation in colorectal cancer. *Human mutation* **21**, 271-276 (2003).
3. Abdel-Fattah G, Yoffe B, Krishnan B, Khaoustov V, Itani K. MDM2/p53 protein expression in the development of colorectal adenocarcinoma. *Journal of gastrointestinal surgery : official journal of the Society for Surgery of the Alimentary Tract* **4**, 109-114 (2000).
4. Khaleghpour K, Li Y, Banville D, Yu Z, Shen SH. Involvement of the PI 3-kinase signaling pathway in progression of colon adenocarcinoma. *Carcinogenesis* **25**, 241-248 (2004).
5. Sheng H, Shao J, Townsend CM, Jr., Evers BM. Phosphatidylinositol 3-kinase mediates proliferative signals in intestinal epithelial cells. *Gut* **52**, 1472-1478 (2003).
6. Itoh N, Semba S, Ito M, Takeda H, Kawata S, Yamakawa M. Phosphorylation of Akt/PKB is required for suppression of cancer cell apoptosis and tumor progression in human colorectal carcinoma. *Cancer-Am Cancer Soc* **94**, 3127-3134 (2002).
7. Guanti G, *et al.* Involvement of PTEN mutations in the genetic pathways of colorectal cancerogenesis. *Hum Mol Genet* **9**, 283-287 (2000).
8. Kojima M, *et al.* Increased nuclear factor-kB activation in human colorectal carcinoma and its correlation with tumor progression. *Anticancer research* **24**, 675-681 (2004).
9. Greten FR, *et al.* IKKbeta links inflammation and tumorigenesis in a mouse model of colitis-associated cancer. *Cell* **118**, 285-296 (2004).
10. Nateri AS, Spencer-Dene B, Behrens A. Interaction of phosphorylated c-Jun with TCF4 regulates intestinal cancer development. *Nature* **437**, 281-285 (2005).
11. Arber N, *et al.* Increased expression of cyclin D1 is an early event in multistage colorectal carcinogenesis. *Gastroenterology* **110**, 669-674 (1996).
12. Zheng H, *et al.* Glycogen synthase kinase-3 beta regulates Snail and beta-catenin expression during Fas-induced epithelial-mesenchymal transition in gastrointestinal cancer. *Eur J Cancer* **49**, 2734-2746 (2013).
13. Castellone MD, Teramoto H, Williams BO, Druey KM, Gutkind JS. Prostaglandin E2 promotes colon cancer cell growth through a Gs-axin-beta-catenin signaling axis. *Science* **310**, 1504-1510 (2005).
14. Wang D, Buchanan FG, Wang H, Dey SK, DuBois RN. Prostaglandin E2 enhances intestinal adenoma growth via activation of the Ras-mitogen-activated protein kinase cascade. *Cancer Res* **65**, 1822-1829 (2005).
15. Sonoshita M, *et al.* Acceleration of intestinal polyposis through prostaglandin receptor EP2 in Apc(Delta 716) knockout mice. *Nat Med* **7**, 1048-1051 (2001).
16. Alexander W. Inhibiting the akt pathway in cancer treatment: three leading candidates. *P & T : a peer-reviewed journal for formulary management* **36**, 225-227 (2011).
17. Semba S, Itoh N, Ito M, Harada M, Yamakawa M. The in vitro and in vivo effects of 2-(4-morpholinyl)-8-phenyl-chromone (LY294002), a specific inhibitor of phosphatidylinositol 3'-kinase, in human colon cancer cells. *Clinical cancer research : an official journal of the American Association for Cancer Research* **8**, 1957-1963 (2002).
18. Rychahou PG, Jackson LN, Silva SR, Rajaraman S, Evers BM. Targeted molecular therapy of the PI3K pathway: therapeutic significance of PI3K subunit targeting in colorectal carcinoma.

Annals of surgery **243**, 833-842; discussion 843-834 (2006).
19. Chu EC, Chai J, Tarnawski AS. NSAIDs activate PTEN and other phosphatases in human colon cancer cells: novel mechanism for chemopreventive action of NSAIDs. *Biochemical and biophysical research communications* **320**, 875-879 (2004).
20. Lee C-E. Activation of p53 and NF-kB degradation by SOCS1 to promote cancer cell apoptosis and to suppress implanted tumor growth. *The Journal of Immunology* **186**, 165.147 (2011).
21. Archer SY, Meng S, Shei A, Hodin RA. p21(WAF1) is required for butyrate-mediated growth inhibition of human colon cancer cells. *Proc Natl Acad Sci U S A* **95**, 6791-6796 (1998).
22. Arber N*, et al.* Antisense to cyclin D1 inhibits the growth and tumorigenicity of human colon cancer cells. *Cancer Res* **57**, 1569-1574 (1997).
23. Hsu CP*, et al.* Anti-interleukin-6 receptor antibody inhibits the progression in human colon carcinoma cells. *European journal of clinical investigation* **41**, 277-284 (2011).
24. Janssen KP*, et al.* Targeted expression of oncogenic K-ras in intestinal epithelium causes spontaneous tumorigenesis in mice. *Gastroenterology* **123**, 492-504 (2002).
25. Subramanian RR, Yamakawa A. Combination therapy targeting Raf-1 and MEK causes apoptosis of HCT116 colon cancer cells. *International journal of oncology* **41**, 1855-1862 (2012).
26. Wilhelm SM*, et al.* BAY 43-9006 exhibits broad spectrum oral antitumor activity and targets the RAF/MEK/ERK pathway and receptor tyrosine kinases involved in tumor progression and angiogenesis. *Cancer Res* **64**, 7099-7109 (2004).
27. Sebolt-Leopold JS*, et al.* Blockade of the MAP kinase pathway suppresses growth of colon tumors in vivo. *Nat Med* **5**, 810-816 (1999).
28. Becker C*, et al.* TGF-beta suppresses tumor progression in colon cancer by inhibition of IL-6 trans-signaling. *Immunity* **21**, 491-501 (2004).
29. Kawamori T*, et al.* Role for sphingosine kinase 1 in colon carcinogenesis. *FASEB journal : official publication of the Federation of American Societies for Experimental Biology* **23**, 405-414 (2009).
30. Ding WX, Ni HM, Chen X, Yu J, Zhang L, Yin XM. A coordinated action of Bax, PUMA, and p53 promotes MG132-induced mitochondria activation and apoptosis in colon cancer cells. *Mol Cancer Ther* **6**, 1062-1069 (2007).
31. Chen JC, Lu KW, Lee JH, Yeh CC, Chung JG. Gypenosides induced apoptosis in human colon cancer cells through the mitochondria-dependent pathways and activation of caspase-3. *Anticancer research* **26**, 4313-4326 (2006).
32. Park HJ, Kim MJ, Ha E, Chung JH. Apoptotic effect of hesperidin through caspase3 activation in human colon cancer cells, SNU-C4. *Phytomedicine : international journal of phytotherapy and phytopharmacology* **15**, 147-151 (2008).

Supplementary Methods

Biological Description of the CAC network

The sustained inflammatory responses in inflammatory bowel disease (IBD) involve an overactivated innate and adaptive immune response[1]. The inflammatory responses can be initiated by antigen-presenting cells such as dendritic cells (DC)[2]. IL-12 (IL12) secreted by activated DCs initiate Th1 immune response, which promotes cell-mediated immunity. Th1 cells (TH1) further mediate the activation of CD8+ cytotoxic T cells (CTL), which play crucial roles in immune surveillance[3]. In addition, Th1 cells also mediate the activation of macrophages (MAC), which are considered as the major source of pro-inflammatory cytokines such as TNF-α (TNFA) and IL-6 (IL6)[2, 4]. DCs also produce the pro-inflammatory cytokine, IL-6, during IBD development[5]. IL-4 (IL4) secreted by DCs initiates humoral immunity by activating Th2 cells (TH2). Th1 responses and Th2 responses counteract each other: Th1 responses are inhibited by Th2 cytokines such as IL-4 and IL-10, while Th2 responses are inhibited by Th1 cytokines such as IFN-γ (IFNG)[6]. DCs also drive the differentiation of regulatory T cells (TREG), resulting in the production of immune suppressive cytokines, TGF-β (TGFB) and IL-10 (IL10) [7, 8], which have been shown to alleviate IBD and lower tumor incidence in CAC cases[9].

The immune microenvironment affects the growth and survival of epithelial cells through cytokines or direct interactions between immune cells and epithelial cells. Meanwhile, cytokines and chemokines produced by epithelial cells, such as IL-6 and CCL2, also take part in shaping the immune microenvironment by regulating immune cells[2, 5, 10]. Pro-inflammatory cytokines, such as TNF-α and IL-6 produced by immune cells, support the proliferation and survival of premalignant intestine epithelial cells (IECs) as well as newly emerged neoplastic cells during chronic inflammation[9]. TNF-α binds to the TNF-α receptor (TNFR) expressed on the cell membrane of IECs and initiates NF-κB signaling. The NF-κB transcription factor up-regulates multiple genes that correlate with cell survival such as Bcl-2 (BCL2), c-Flip (CFLIP), c-IAPs (IAP) and XIAP[11]. However, upon activation, TNFR also recruits FADD and initiates caspase-8 (CASP8) dependent apoptotic cell death, which can be blocked by c-Flip[12]. In addition, the activation of TNFR couples with several cellular processes such as production of ROS, up-regulation of COX2 and activating the JNK pathway[12, 13, 14]. Except for TNF-α, IL-6 signaling has also been proven to play important roles in IBDs and CAC development[15]. Activation of gp130 by IL-6 leads to the phosphorylation and nuclear translocation of STAT3 transcription factor[15]. Similar to NF-κB, STAT3 enhances cell survival by up-regulating Bcl-2 and c-IAPs[11]. STAT3 can also promote cell proliferation by driving the expression of Cyclin D1 (CYCLIND1)[16]. Except for signaling through STAT3, gp130 also activates ERK MAPK signaling cascade to promote cell proliferation[17].

In addition to the promoting effect on cell survival and proliferation, the immune microenvironment also exerts anti-proliferation and pro-apoptosis effect on IECs. TGF-β secreted by immune cells not only suppresses the inflammatory response, but also suppresses cell proliferation through TGF-β/SMADs signaling[18]. Cytotoxic T lymphocytes (CTL) activated by Th1 cells express Fas ligand (FASL) and initiate Fas mediated apoptosis of epithelial cells[19]. This process also involves the activation of caspase-8 and can be blocked by c-Flip[20].

Except for those four pathways (TNF-α/NF-κB, IL-6/STAT3, TGF-β/SMADs, and FasL/Fas) that

mediate the crosstalk between immune microenvironment and epithelial cells, recent studies also emphasize sphingolipid signaling in inflammation and cancer development[5]. Binding of TNF-α to TNFR activates SPHK1, which is a key regulator of sphingolipid metabolism. SPHK1 produces sphingosine 1-phosphate (S1P), another bioactive sphingolipid metabolite that has been shown to correlate with cell proliferation and survival. S1P is also a cofactor of TRAF2 and plays an essential role in initiating NF-κB signaling[21]. SPHK1 activation also decreases the cellular level of another sphingolipid metabolite, ceramide, which plays an opposite role to S1P[22]. Moreover, TNF-α signaling also leads to the generation of ceramide by activating sphingomyelinase[23, 24]. Ceramide inhibits cell proliferation and induces apoptosis through directly binding and activating PP2A and MEKK1. Ceramide has also been shown to directly trigger mitochondrial outer membrane permeabilization (MOMP), leading to the release of pro-apoptotic proteins such as cytochrome C (CYTC) and Smac (SMAC)[25]. These proteins are able to initiate mitochondrial apoptosis pathways, which involves the sequential activation of caspase-9 (CASP9) and caspase-3 (CASP3)[25]. Further , PGE2, another lipid signaling molecule, has been shown to play important roles in CAC[26]. PGE2 binds to the EP2 receptor on epithelial cells and activates RAS/RAF/MEK/ERK signaling as well as PI3K/AKT signaling, two pathways that tightly correlate with cell growth and survival[27, 28]. In addition, activated EP2 leads to the phosphorylation and inhibition of glycogen synthase kinase-3β (GSK3B) and therefore promotes β-catenin signaling[29]. Increased activity of β-catenin has been observed in both CAC and sporadic colon cancers[9].

Detailed explanation of the Boolean rules of the CAC network.

TNFA* = MAC
TNFR* = TNFA
FADD* = TNFR or FAS
IKK* = AKT or (S1P and TNFR)
IKB* = not IKK
NFKB* = not IKB
FAS* = CTL
COX2* = S1P and TNFR
PGE2* = COX2
EP2* = PGE2
S1P* = SPHK1
SPHK1* = ERK or TNFR

In IBDs such as Crohn's disease and ulcerative colitis, activated macrophages are the major source of TNF-α[4]. TNF-α binds to TNF-α receptors and activates their downstream target tumor necrosis factor receptor 1-associated death domain (TRADD)[12]. On one hand, the interaction between TRADD and Fas-Associated protein with Death Domain (FADD) stimulates the activation of caspase-8 (represented as CASP8), which is the initiator caspase of the death receptor pathway of apoptosis[12]. Binding of Fas ligand expressed on CTL to Fas on epithelial cells or tumor cells can also recruit and activate FADD[19]. On the other hand, TRADD recruits TNF receptor-associated factor 2 (TRAF2), which can in turn recruit and

activate IκB kinase (IKK) and initiate NF-κB signaling. In unstimulated cells, NF-κB dimers (represented as NFKB) are sequestered in cytoplasm by a family of inhibitors called IκB (represented by "IKB"). The activated IKK leads to the phosphorylation and subsequent degradation of IκB, which enables the nuclear translocation and activation of NF-κB transcription factor[12]. TNFR, TRADD and TRAF2 are collapsed into "TNFR" in the Boolean rules. A recent study identified S1P as a missing cofactor of TRAF2 and showed S1P played a key role in canonical NF-κB activation[21]. Therefore, an "AND" is used between TNFR and S1P to indicate their synergistic positive effect on IKK in the Boolean rules. In addition, NF-κB can be activated through PI3K/AKT/IKK pathway independent of TNF-α signaling[30, 31]. Regulation of cyclooxygenase-2 (COX2) expression and PGE_2 (represented by "PGE2") production by the SPHK1/S1P pathway plays an important role in colon carcinogenesis[32]. As the up-regulation of COX2 by S1P may be TNF-α dependent, an "AND" is used to link "S1P" and "TNFR". The actions of PGE2 are mediated by signaling through four G protein-coupled receptors EP_1, EP_2, EP_3 and EP_4 (represented by "EP2")[33]. Although two isoforms of sphingosine kinase (SPHK1 and SPHK2) are responsible for the generation of S1P, SPHK1 is considered as the major source of S1P during colitis and colon carcinogenesis[5, 34]. SPHK1 can be recruited and activated by TNF receptor[21, 35] and it can also be phosphorylated and activated by Erk1/2 (ERK)[36].

IL6* = MAC or DC or NFKB
GP130* = IL6
JAK* = GP130 and not SOCS
STAT3* = JAK
SOCS* = STAT3
RAS* = EP2 or GP130
RAF* = CERAMIDE or RAS
MEK* = RAF or ROS
ERK* = MEK
FOS* = ERK
PI3K* = (EP2 or RAS) and not PTEN
PTEN* = P53 and not (NFKB or JUN)
AKT* = PI3K and not (PP2A or CASP3)
PP2A* = CERAMIDE and not AKT
CERAMIDE* = SMASE and not SPHK1
SMASE* = P53 or FADD

Macrophage and dendritic cells act as the major source of IL-6 (represented by "IL6") during the development of CAC[2, 5]. Meantime, IECs also produce IL-6 through the activation of NF-κB[2, 5]. The interaction between IL-6 and its receptor triggers the gp130 and IL-6R proteins to form a complex (represented by "GP130"), resulting in the activation of the associated Janus kinase 2 (JAK2, represented as "JAK"). This leads to the subsequent phosphorylation and dimerization of Signal transducer and activator of transcription 3 (STAT3). Then the dimerized STAT3 protein translocates into cell nucleus and activates the transcription of its targets genes. Suppressor of cytokine signaling proteins (SOCS) are well-known STAT3

target genes, which are also involved in inhibiting JAK-STAT signaling, thus forming a negative feed-back loop in the CAC network. In addition, activated gp130 or PGE_2 also leads to the activation of ERK MAPK signaling (RAS/RAF/MEK/ERK)[17, 37], resulting in the activation of proto-oncogene c-Fos (represented by "FOS")[38]. Raf kinase (RAF) within the ERK/MAPK pathway has been shown to be directly phosphorylated and activated by ceramide-activated protein (CAP) kinase ("CERAMIDE" and "CAPK" are collapsed in to "CERAMIDE")[39]. Reactive oxygen species (ROS) are also well known to induce the activation of the ERK/MAPK pathway. This action occurs on an upstream regulator of ERK1/2, as the inhibitors (U0126 and PD98059) of MEK1 and 2 both blocked the oxidative stress-induced ERK1/2 activation[40].

PGE_2 activates the of PI3K/AKT pathway through EP2 or EP4 receptors[27]. PI3Ks can also be directly stimulated by GTP-bound Ras[41] and activation of the PI3K/AKT pathway via K-ras mutations is considered one of the most common mechanisms involved in colorectal carcinogenesis[42]. PTEN protein acts as a phosphatase to dephosphorylate phosphatidylinositol (3,4,5)-trisphosphate (PIP3) and thus suppresses PI3K activation[43]. PTEN is a tumor suppressor gene that is positively regulated by p53[44]and negatively regulated by NF-κB[45]. Activation of c-Jun, a component of the AP-1 family of transcription factor, also leads to the down-regulation of PTEN gene[46]. Activated Akt (AKT) can be inhibited through dephosphorylation by Protein phosphatase 2 (PP2A)[47] or direct cleavage by caspase-3[48]. PP2A can be negatively regulated by mammalian target of rapamycin (mTOR)[49, 50], which is activated by the PI3K/AKT pathway (mTOR and its direct upstream regulator AKT are collapsed into "AKT"). While ceramides stimulate PP2A directly or through inhibiting an endogenous PP2A inhibitor, I2PP2A[51, 52]. Ceramides are generated by sphingomyelinases (represented by "SMASE"), which directly hydrolyse sphingomyelins into ceramides. Neutral sphingomyelinases can be activated by P53 while acid sphingomyelinases are activated by TNF receptors through FADD[23, 24]. As ceramides are the precursors of S1P, SPHK1 also tightly regulates the cellular level of ceramides[22].

CASP8* = FADD and not (CFLIP or P21)
CFLIP* = NFKB
TBID* = CASP8 and not BCL2
BAX* = ((TBID or P53) and PP2A) and not AKT
BCL2* = (STAT3 or NFKB) and not (P53 or PP2A)
CYTC* = MOMP
SMAC* = MOMP
MOMP* = (BAX or TBID or CERAMIDE) and not BCL2
CASP3* = (CASP8 or CASP9) and not IAP
CASP9* = CYTC and not (IAP or P21)
IAP* = (NFKB or STAT3) and not SMAC
Apoptosis* = CASP3

The extrinsic apoptosis pathway is initiated by the activation of caspase-8 (represented by "CASP8"). However, the activation of caspase-8 can be inhibited by p21/WAF1 (represented by "P21")[53] or c-FLIP (represented by "CFLIP")[20], a downstream target of NF-κB(NFKB)[54].

Activated caspase-8 cleavages BID and generates truncated BID (tBID, represented by "TBID"), which in turn mediates the mitochondria damage such as mitochondrial outer membrane permeabilization (MOMP)[55]. MOMP can also be induced by the intrinsic pathway which involves the dynamic balance of Bcl-2 family of proteins. The pro-apoptotic members of this family, such as Bax and Bak (represented by "BAX"), can trigger MOMP while the anti-apoptotic members, such as Bcl-2 and Bcl-xL (represented by "BCL2"), prevent MOMP[56]. The pro-apoptotic sphingolipid ceramide (CERAMIDE), can also trigger MOMP through forming ceramide channels or other mechanisms[57]. P53 activates Bax through up-regulating bax gene expression or by directly associating with Bax protein[58, 59]. Bax and Bak can also be activated by tBID[60]. The phosphorylation of Bax by Akt (AKT) inhibits Bax effects on the mitochondria by maintaining the protein in the cytoplasm, heterodimerized with anti-apoptotic Bcl-2 family members[61]. Since the dephosphorylation of Bax by PP2A is required for its effect on mitochondria, an "AND" is used between PP2A and "P53 or TBID"[62]. The occurrence of MOMP leads to the release of pro-apoptotic proteins such as Smac/DIABLO (SMAC) and cytochrome c (represented by "CYTC")[63]. Cytosolic cytochrome c binds to Apaf-1 and triggers the formation of the apoptosome, which mediates the activation of caspase-9 (represented by "CASP9"), which is inhibited by P21[53]. Activated caspase-9 cleaves and activates downstream executioner caspases such as caspase-3 (represented by "CASP3")[64], which can also be cleaved and activated by caspase-8[64]. The activation of executioner caspases such as caspase-3 initiates the apoptosis event and is generally considered as "a point of no return"[65]. Members of the Inhibitors of Apoptosis (IAPs) family, such as XIAP and cIAPs (represented by "IAP"), inhibit the activity of caspase-3 and caspase-9[63]. IAPs are positively regulated by both STAT3 and NF-κB[11] while they are inhibited by Smac/DIABLO through direct binding[63]. STAT3 and NF-κB also enhance cell survival through up-regulating anti-apoptotic Bcl-2 family members such as Bcl-2 and Bcl-xL (represented as "BCL2"). On the other hand, Bcl-2 family members have been shown to be down-regulated by P53[66] and can be inhibited through dephosphorylation by PP2A[67].

ROS* = TNFR and not SOD
SOD* = NFKB or STAT3
ASK1* = ROS and not P21
JNK* = ASK1 or MEKK1
MEKK1* = CERAMIDE or TGFR or TNFR
JUN* = ((BCATENIN or ERK) and JNK) and not GSK3B
GSK3B* = not (EP2 or AKT)
BCATENIN* = not (GSK3B and APC)
CYCLIND1* = (BCATENIN or STAT3 or JUN) and not GSK3B
Proliferation* = (FOS and CYCLIND1) and not (P21 or CASP3)

Reactive oxygen species (ROS) can be generated by TNF through activated TNF receptors[13]. NF-κB and STAT3 signaling up-regulates superoxide dismutases (represented by "SOD"), which eliminates ROS rapidly[11]. Accumulated ROS activates Apoptosis signal-regulating kinase 1 (ASK1) and leads to the subsequent activation of c-Jun N-terminal kinases

(represented by “JNK”)[68]. Meanwhile, ASK1 activation can be inhibited by P21 through direct binding[68]. JNKs can also be activated by extracellular signal-regulated kinase kinase kinase 1 (MEKK1), which is activated by various stimulus such as ceramide[69], TGF receptors (TGFR)[70] and TNF receptors (TNFR)[12]. JNKs promote intestinal carcinogenesis through their ability to phosphorylate c-jun and to activate AP1[71, 72]. Studies have also shown that the activation of c-jun could be facilitated by ERK/MAPK pathway[73] or β-catenin/TCF4 signaling[71]. An “AND” is then used between JNK and other activators since the initial phosphorylation of c-jun by JNKs is considered essential for the transcriptional activity of c-jun and AP1[30]. The transcriptional activity of c-jun is inhibited through phosphorylation by glycogen synthase kinase-3 (represented as “GSK3B”). GSK3-β plays a key role in regulating Wnt/β-catenin signaling. GSK3-β normally forms a complex with axin and adenomatous polyposis coli (represented by “APC”), which sequesters β-catenin in the cytoplasm[74]. The phosphorylation of β-catenin by GSK3-β leads to its ubiquitination and subsequent degradation by cellular proteosomes, thus preventing the nuclear translocation of β-catenin and the activation of its target gene, cyclin D1 (represented by “CYCLIND1”)[75]. Either inhibition of GSK3-β, by PGE2 signaling[29] or Akt/PKB pathway[76], or inactive mutation of APC results in the increased activation of β-catenin, which has been commonly observed in colon cancers[77]. Except for β-catenin, the activation of STAT3[16] or c-jun[78] transcription factors also leads to the up-regulation of cyclin D1, which is required for cell cycle progression and cell proliferation. Cyclin D1 has also been shown to be inhibited by GSK3-β through direct phosphorylation[79]. The proliferation state can be inhibited by the activation of caspase-3 or P21, which can induce apoptosis and cell cycle arrest respectively.

P53* = (PTEN or JNK or ATM) and not MDM2
MDM2* = (P53 and AKT) and not (GSK3B or ATM)
ATM* = ROS
P21* = (P53 or SMAD) and not (GSK3B or CASP3)
SMAD* = TGFR and not JUN
TGFR* = TGFB and not SMAD7
SMAD7* = SMAD or NFKB

Mutations of the tumor suppressor gene TP53 and its product p53 (represented by “P53) have been widely observed in sporadic colon cancer as well as CAC. P53 and its down-stream target MDM2 form a negative feedback loop that keeps the level of p53 low in the absence of p53-stabilizing signals[80]. Although the MDM2 gene can be up-regulated by P53, the phosphorylation of MDM2 by Akt/PKB (AKT) is required for the nuclear translocation of MDM2 and its p53 inhibiting activity[81, 82]. Therefore, an “AND” is used between “AKT” and “P53”. The phosphorylation of MDM2 by ATM or GSK3-β has been shown to attenuate MDM2 mediated P53 ubiquitination and degradation[83, 84, 85]. P53 can be phosphorylated and activated by Ataxia telangiectasia mutated (ATM), which senses DNA damage caused by oxidative stress or other factors[86]. Oxidative stresses, such as the increased level of ROS, also activates ATM directly[87]. Stress-induced phosphorylation of p53 by JNKs leads to the stabilization of p53[88], which plays a key role in regulating the apoptosis of colon carcinoma cells[89]. In addition, PTEN has also been shown to associate with p53 directly and enhances its

transactivation in a phosphatase-independent manner[90, 91]. $P21^{Waf1/Cip1}$ (represented by P21) is a p53 target gene and is necessary for p53 mediated cell cycle arrest[92, 93]. The phosphorylation of $p21^{Waf1/Cip1}$ by GSK-3β promotes its degradation[94, 95] and it can also be cleaved by executioner caspases, such as caspase-3 in various cell types, including colon carcinoma cells[96, 97]. In addition, TGF-β signaling through receptor-regulated Smads (R-SMADs, represented by "SMAD") also induces $p21^{Waf1/Cip1}$ expression and leads to the growth arrest of IECs[98]. TGF-β mediated transcriptional activity of R-SMADs can be down-regulated by c-Jun[99]. The inhibitory Smads (I-SMADs), such as SMAD7, can be up-regulated by R-SMADs[100]. However, SMAD7 inhibits the TGF-β mediated activation of R-SMADs and therefore forms another negative feedback loop in the CAC network[100, 101]. In addition, NF-κB has also been shown to suppress the TGF-β/Smad pathway by transcriptional activation of the inhibitory Smad7[102].

TH1* = (IL12 or IFNG) and not (IL10 or TGFB or IL4)
IL12* = DC or MAC
TH2* = IL4 and not (IFNG or TGFB)
IL4* = DC or TH2
IFNG* = TH1 or CTL
CTL* = IFNG and not TGFB
MAC* = (IFNG or CCL2) and not IL10
TREG* = (IL10 or DC) and not IL6
TGFB* = TREG
IL10* = TREG or TH2
CCL2* = NFKB
DC* = (CCL2 or TNFA) and not IL10

Various immune cells, cytokines and chemokines take part in the different periods during the development of IBDs as well as CAC progress. As potent antigen-presenting cells, intestine dendritic cells (DCs, represented by "DC") play key roles in inflammatory responses in IBDs[2]. Dendritic cells recruit different types of immune cells depending on the cytokine microenvironment. IL-12 (represented by "IL12") secreted by DCs activates type 1 T helper cells (Th1 helper cells, represented by "TH1") and initiates Th1 immune response, which promotes cell-mediated immunity[103]. Activated Th1 cells produce interferon-γ (IFN-γ, represented by "IFNG"), which activates macrophages (represented by "MAC")[3, 104]. IFN-γ is also able to up-regulate macrophage IL-12 production, and this enhanced IL-12/IFN-γ loop is likely to be involved in the chronic inflammation within the intestine[3]. In addition, Th1 cells mediate the activation of CD8+ cytotoxic T lymphocytes (represented by "CTL") through the production of IL-2 and IFN-γ (IL2 and IFNG are collapsed into "IFNG")[105]. The activated CTLs can in turn act as another source of IFN-γ[106]. On the other hand, IL-4 (IL-4, represented as "IL4") secreted by dendritic cells guides the maturation of type 2 T helper cells (Th2 cells , represented by "TH2"), which initiate humoral immunity[106, 107]. Th1 responses and Th2 responses counteract each other: Th1 responses are inhibited by Th2 cytokines such as IL-4 and IL-10, while Th2 responses are inhibited by Th1 cytokines such as IFN-γ[6]. DCs can also drive the differentiation of regulatory T cells (represented by "TREG"), which produce

immune suppressive cytokines, TGF-β (represented by "TGFB") and IL-10 (represented by "IL10")[7, 8]. IL-10 can inhibit the pro-inflammatory functions of a broad array of immune cells, such as Th1 cells, macrophages and DCs[8, 108, 109]. TGF-β secreted by Tregs inhibits both Th1 and Th2 responses and it can also potently inhibit immune surveillance mediated by CD8+ cytotoxic T lymphocytes[110, 111] . Excessive IL-6 has been shown to suppress differentiation of Treg cells from naïve T cells[112]. Myeloid-derived immune cells such as macrophages and DCs have been shown to be recruited and activated by chemokine (C-C motif) ligand 2 (CCL2), which can be produced by IECs upon NF-κB activation during inflammatory responses[10, 113, 114]. In addition to CCL2, TNF-α present in the microenvironment also enhances DC survival and maturation[115, 116].

Immunoblot analysis

Cells were harvested in PIRA lysis buffer (50 mM Tris, 150 mM NaCl, 1% Triton X-100, 0.1% SDS and 1% sodium deoxycholate, with protease and phosphatase inhibitors). Protein concentrations were determined using a BCA assay (Thermoscientific). Proteins were subjected to SDS-PAGE, transferred to PVDF membranes (Immobilon-P, Millipore) and blocked for 1 h at room temperature with 5% milk in 1 x TBST. Membranes were incubated overnight with antibodies as indicated and exposed to secondary HRP-conjuncted anti-mouse or anti-rabbit antibodies at 1:2000 to 1:5,000, respectively, for 1 h at room temperature. The antigen-antibody reaction was visualised with an enhanced chemiluminescence assay (Thermoscientific) or Femto chemiluminescence assay (Thermoscientific). The results shown are representative of at least three independent experiments.

Cell viability assays

HT29 cells were seeded in 96-well plates at densities of 3×10^3 cells per well. The following day, the cells were rinsed, and fresh medium was added with DMSO or the various indicated inhibitors for 48 h. Cell viability were assayed using sulforhodamine B (SRB). Briefly, the culture medium was aspirated, and RPMI 1640 medium with 10% trichloroacetic acid (TCA) was added to each well and allowed to stand for 24 h at 4°C to precipitate the proteins. Then, the precipitated proteins were stained for 15 min at room temperature with 0.4% (w/v) SRB in an acetic acid solution 1% v/v, washed with 1% acetic acid 5 times and then dried. The adherent SRB was solubilised in 10 mmol/l Tris buffer. Finally, the absorbance (optical density, OD) was read at a wavelength of 560 nm on an ELISA plate reader. Cell viability was measured as survival rate, which was calculated as follows: (OD treated/OD control) × 100%.

siRNA

siRNAs were ordered as RP-HPLC-purified duplexes from Sigma-Aldrich. The sequences were as follows:
si-AKT1-#1: GUGCCAUGAUCUGUAUUUdTdT (sense);
si-AKT1-#2: GAGACUGACACCAGGUAUUdTdT (sense);
si-AKT2-#1: CUCUUCGAGCUCAUCCUCAdTdT (sense);

si-AKT2-#2: CAGAAUGCCAGCUGAUGAAdTdT (sense);
si-AKT3-#1: GAAAGAUUGUGUACCGUGAdTdT (sense); and
si-AKT3-#2: GACAUUAAAUUUCCUCGAAdTdT (sense).

References for Supplementary Methods:

1. Xavier RJ, Podolsky DK. Unravelling the pathogenesis of inflammatory bowel disease. *Nature* 448, 427-434 (2007).
2. Hart AL*, et al.* Characteristics of intestinal dendritic cells in inflammatory bowel diseases. *Gastroenterology* 129, 50-65 (2005).
3. Pallone F, Monteleone G. Interleukin 12 and Th1 responses in inflammatory bowel disease. *Gut* 43, 735-736 (1998).
4. Braegger CP, MacDonald TT. Immune mechanisms in chronic inflammatory bowel disease. *Annals of allergy* 72, 135-141 (1994).
5. Liang J*, et al.* Sphingosine-1-Phosphate Links Persistent STAT3 Activation, Chronic Intestinal Inflammation, and Development of Colitis-Associated Cancer. *Cancer Cell* 23, 107-120 (2013).
6. Wilczynski JR. Th1/Th2 cytokines balance--yin and yang of reproductive immunology. *Eur J Obstet Gynecol Reprod Biol* 122, 136-143 (2005).
7. Zou T, Caton AJ, Koretzky GA, Kambayashi T. Dendritic cells induce regulatory T cell proliferation through antigen-dependent and -independent interactions. *J Immunol* 185, 2790-2799 (2010).
8. Huibregtse IL, van Lent AU, van Deventer SJ. Immunopathogenesis of IBD: insufficient suppressor function in the gut? *Gut* 56, 584-592 (2007).
9. Terzic J, Grivennikov S, Karin E, Karin M. Inflammation and colon cancer. *Gastroenterology* 138, 2101-2114 e2105 (2010).
10. Popivanova BK*, et al.* Blockade of a chemokine, CCL2, reduces chronic colitis-associated carcinogenesis in mice. *Cancer Res* 69, 7884-7892 (2009).
11. Grivennikov SI, Karin M. Dangerous liaisons: STAT3 and NF-kappaB collaboration and crosstalk in cancer. *Cytokine Growth Factor Rev* 21, 11-19 (2010).
12. Chen G, Goeddel DV. TNF-R1 signaling: a beautiful pathway. *Science* 296, 1634-1635 (2002).
13. Yamauchi N, Kuriyama H, Watanabe N, Neda H, Maeda M, Niitsu Y. Intracellular hydroxyl radical production induced by recombinant human tumor necrosis factor and its implication in the killing of tumor cells in vitro. *Cancer Res* 49, 1671-1675 (1989).
14. Medeiros R*, et al.* The role of TNF-alpha signaling pathway on COX-2 upregulation and cognitive decline induced by beta-amyloid peptide. *Behav Brain Res* 209, 165-173 (2010).
15. Grivennikov S*, et al.* IL-6 and Stat3 are required for survival of intestinal epithelial cells and development of colitis-associated cancer. *Cancer Cell* 15, 103-113 (2009).
16. Corvinus FM*, et al.* Persistent STAT3 activation in colon cancer is associated with enhanced cell proliferation and tumor growth. *Neoplasia* 7, 545-555 (2005).
17. Heinrich PC, Behrmann I, Haan S, Hermanns HM, Muller-Newen G, Schaper F. Principles of interleukin (IL)-6-type cytokine signalling and its regulation. *The Biochemical journal* 374, 1-20 (2003).
18. Bierie B, Moses HL. Transforming growth factor beta (TGF-beta) and inflammation in cancer. *Cytokine Growth Factor Rev* 21, 49-59 (2010).

19. Pinkoski MJ, Brunner T, Green DR, Lin T. Fas and Fas ligand in gut and liver. *American journal of physiology Gastrointestinal and liver physiology* 278, G354-366 (2000).
20. Kataoka T. The caspase-8 modulator c-FLIP. *Crit Rev Immunol* 25, 31-58 (2005).
21. Alvarez SE*, et al.* Sphingosine-1-phosphate is a missing cofactor for the E3 ubiquitin ligase TRAF2. *Nature* 465, 1084-1088 (2010).
22. Shida D, Takabe K, Kapitonov D, Milstien S, Spiegel S. Targeting SphK1 as a new strategy against cancer. *Curr Drug Targets* 9, 662-673 (2008).
23. Wiegmann K, Schwandner R, Krut O, Yeh WC, Mak TW, Kronke M. Requirement of FADD for tumor necrosis factor-induced activation of acid sphingomyelinase. *J Biol Chem* 274, 5267-5270 (1999).
24. Sawai H, Hannun YA. Ceramide and sphingomyelinases in the regulation of stress responses. *Chem Phys Lipids* 102, 141-147 (1999).
25. Pettus BJ, Chalfant CE, Hannun YA. Ceramide in apoptosis: an overview and current perspectives. *Biochim Biophys Acta* 1585, 114-125 (2002).
26. Kawai N, Tsujii M, Tsuji S. Cyclooxygenases and colon cancer. *Prostaglandins Other Lipid Mediat* 68-69, 187-196 (2002).
27. Castellone MD, Teramoto H, Williams BO, Druey KM, Gutkind JS. Prostaglandin E2 promotes colon cancer cell growth through a Gs-axin-beta-catenin signaling axis. *Science* 310, 1504-1510 (2005).
28. Regan JW. EP2 and EP4 prostanoid receptor signaling. *Life Sci* 74, 143-153 (2003).
29. Shao J, Jung C, Liu C, Sheng H. Prostaglandin E2 Stimulates the beta-catenin/T cell factor-dependent transcription in colon cancer. *J Biol Chem* 280, 26565-26572 (2005).
30. Agarwal A*, et al.* The AKT/I kappa B kinase pathway promotes angiogenic/metastatic gene expression in colorectal cancer by activating nuclear factor-kappa B and beta-catenin. *Oncogene* 24, 1021-1031 (2005).
31. Shant J, Cheng K, Marasa BS, Wang JY, Raufman JP. Akt-dependent NF-kappaB activation is required for bile acids to rescue colon cancer cells from stress-induced apoptosis. *Experimental cell research* 315, 432-450 (2009).
32. Kawamori T*, et al.* Sphingosine kinase 1 is up-regulated in colon carcinogenesis. *FASEB journal : official publication of the Federation of American Societies for Experimental Biology* 20, 386-388 (2006).
33. Breyer RM, Bagdassarian CK, Myers SA, Breyer MD. Prostanoid receptors: subtypes and signaling. *Annual review of pharmacology and toxicology* 41, 661-690 (2001).
34. Kawamori T*, et al.* Role for sphingosine kinase 1 in colon carcinogenesis. *FASEB journal : official publication of the Federation of American Societies for Experimental Biology* 23, 405-414 (2009).
35. Snider AJ*, et al.* A role for sphingosine kinase 1 in dextran sulfate sodium-induced colitis. *FASEB journal : official publication of the Federation of American Societies for Experimental Biology* 23, 143-152 (2009).
36. Pitson SM*, et al.* Activation of sphingosine kinase 1 by ERK1/2-mediated phosphorylation. *EMBO J* 22, 5491-5500 (2003).
37. Wang D, Buchanan FG, Wang H, Dey SK, DuBois RN. Prostaglandin E2 enhances intestinal adenoma growth via activation of the Ras-mitogen-activated protein kinase cascade. *Cancer Res* 65, 1822-1829 (2005).

38. Fang JY, Richardson BC. The MAPK signalling pathways and colorectal cancer. *Lancet Oncol* 6, 322-327 (2005).
39. Yao B, Zhang Y, Delikat S, Mathias S, Basu S, Kolesnick R. Phosphorylation of Raf by ceramide-activated protein kinase. *Nature* 378, 307-310 (1995).
40. McCubrey JA*, et al.* Roles of the Raf/MEK/ERK pathway in cell growth, malignant transformation and drug resistance. *Biochim Biophys Acta* 1773, 1263-1284 (2007).
41. Rodriguez-Viciana P*, et al.* Phosphatidylinositol-3-OH kinase as a direct target of Ras. *Nature* 370, 527-532 (1994).
42. Ikenoue T*, et al.* Functional analysis of PIK3CA gene mutations in human colorectal cancer. *Cancer Res* 65, 4562-4567 (2005).
43. Carnero A, Blanco-Aparicio C, Renner O, Link W, Leal JF. The PTEN/PI3K/AKT signalling pathway in cancer, therapeutic implications. *Current cancer drug targets* 8, 187-198 (2008).
44. Stambolic V*, et al.* Regulation of PTEN transcription by p53. *Mol Cell* 8, 317-325 (2001).
45. Kim S, Domon-Dell C, Kang J, Chung DH, Freund JN, Evers BM. Down-regulation of the tumor suppressor PTEN by the tumor necrosis factor-alpha/nuclear factor-kappaB (NF-kappaB)-inducing kinase/NF-kappaB pathway is linked to a default IkappaB-alpha autoregulatory loop. *J Biol Chem* 279, 4285-4291 (2004).
46. Hettinger K*, et al.* c-Jun promotes cellular survival by suppression of PTEN. *Cell Death Differ* 14, 218-229 (2007).
47. Trotman LC, Alimonti A, Scaglioni PP, Koutcher JA, Cordon-Cardo C, Pandolfi PP. Identification of a tumour suppressor network opposing nuclear Akt function. *Nature* 441, 523-527 (2006).
48. Jahani-Asl A, Basak A, Tsang BK. Caspase-3-mediated cleavage of Akt: involvement of non-consensus sites and influence of phosphorylation. *FEBS letters* 581, 2883-2888 (2007).
49. Hartley D, Cooper GM. Role of mTOR in the degradation of IRS-1: regulation of PP2A activity. *J Cell Biochem* 85, 304-314 (2002).
50. Hay N, Sonenberg N. Upstream and downstream of mTOR. *Genes & development* 18, 1926-1945 (2004).
51. Mukhopadhyay A*, et al.* Direct interaction between the inhibitor 2 and ceramide via sphingolipid-protein binding is involved in the regulation of protein phosphatase 2A activity and signaling. *FASEB journal : official publication of the Federation of American Societies for Experimental Biology* 23, 751-763 (2009).
52. Dobrowsky RT, Kamibayashi C, Mumby MC, Hannun YA. Ceramide activates heterotrimeric protein phosphatase 2A. *J Biol Chem* 268, 15523-15530 (1993).
53. Gartel AL, Tyner AL. The role of the cyclin-dependent kinase inhibitor p21 in apoptosis. *Mol Cancer Ther* 1, 639-649 (2002).
54. Micheau O, Lens S, Gaide O, Alevizopoulos K, Tschopp J. NF-kappaB signals induce the expression of c-FLIP. *Mol Cell Biol* 21, 5299-5305 (2001).
55. Li H, Zhu H, Xu CJ, Yuan J. Cleavage of BID by caspase 8 mediates the mitochondrial damage in the Fas pathway of apoptosis. *Cell* 94, 491-501 (1998).
56. Tsujimoto Y. Role of Bcl-2 family proteins in apoptosis: apoptosomes or mitochondria? *Genes Cells* 3, 697-707 (1998).
57. Siskind LJ. Mitochondrial ceramide and the induction of apoptosis. *J Bioenerg Biomembr* 37, 143-153 (2005).

58. Miyashita T, Reed JC. Tumor suppressor p53 is a direct transcriptional activator of the human bax gene. *Cell* 80, 293-299 (1995).
59. Chipuk JE*, et al.* Direct activation of Bax by p53 mediates mitochondrial membrane permeabilization and apoptosis. *Science* 303, 1010-1014 (2004).
60. Kim H*, et al.* Stepwise activation of BAX and BAK by tBID, BIM, and PUMA initiates mitochondrial apoptosis. *Mol Cell* 36, 487-499 (2009).
61. Gardai SJ*, et al.* Phosphorylation of Bax Ser184 by Akt regulates its activity and apoptosis in neutrophils. *J Biol Chem* 279, 21085-21095 (2004).
62. Xin M, Deng X. Protein phosphatase 2A enhances the proapoptotic function of Bax through dephosphorylation. *J Biol Chem* 281, 18859-18867 (2006).
63. Wang X. The expanding role of mitochondria in apoptosis. *Genes & development* 15, 2922-2933 (2001).
64. Stennicke HR*, et al.* Pro-caspase-3 is a major physiologic target of caspase-8. *J Biol Chem* 273, 27084-27090 (1998).
65. Kroemer G, Reed JC. Mitochondrial control of cell death. *Nat Med* 6, 513-519 (2000).
66. Miyashita T*, et al.* Tumor suppressor p53 is a regulator of bcl-2 and bax gene expression in vitro and in vivo. *Oncogene* 9, 1799-1805 (1994).
67. Garcia A*, et al.* Serine/threonine protein phosphatases PP1 and PP2A are key players in apoptosis. *Biochimie* 85, 721-726 (2003).
68. Matsukawa J, Matsuzawa A, Takeda K, Ichijo H. The ASK1-MAP kinase cascades in mammalian stress response. *J Biochem* 136, 261-265 (2004).
69. Huwiler A, Xin C, Brust AK, Briner VA, Pfeilschifter J. Differential binding of ceramide to MEKK1 in glomerular endothelial and mesangial cells. *Biochim Biophys Acta* 1636, 159-168 (2004).
70. Derynck R, Zhang YE. Smad-dependent and Smad-independent pathways in TGF-beta family signalling. *Nature* 425, 577-584 (2003).
71. Sancho R*, et al.* JNK signalling modulates intestinal homeostasis and tumourigenesis in mice. *EMBO J* 28, 1843-1854 (2009).
72. Davis RJ. Signal transduction by the JNK group of MAP kinases. *Cell* 103, 239-252 (2000).
73. Vial E, Marshall CJ. Elevated ERK-MAP kinase activity protects the FOS family member FRA-1 against proteasomal degradation in colon carcinoma cells. *J Cell Sci* 116, 4957-4963 (2003).
74. Valvezan AJ, Zhang F, Diehl JA, Klein PS. Adenomatous polyposis coli (APC) regulates multiple signaling pathways by enhancing glycogen synthase kinase-3 (GSK-3) activity. *J Biol Chem* 287, 3823-3832 (2012).
75. Tetsu O, McCormick F. Beta-catenin regulates expression of cyclin D1 in colon carcinoma cells. *Nature* 398, 422-426 (1999).
76. Cross DA, Alessi DR, Cohen P, Andjelkovich M, Hemmings BA. Inhibition of glycogen synthase kinase-3 by insulin mediated by protein kinase B. *Nature* 378, 785-789 (1995).
77. Morin PJ*, et al.* Activation of beta-catenin-Tcf signaling in colon cancer by mutations in beta-catenin or APC. *Science* 275, 1787-1790 (1997).
78. Shaulian E, Karin M. AP-1 in cell proliferation and survival. *Oncogene* 20, 2390-2400 (2001).
79. Diehl JA, Cheng M, Roussel MF, Sherr CJ. Glycogen synthase kinase-3beta regulates cyclin D1 proteolysis and subcellular localization. *Genes & development* 12, 3499-3511 (1998).

80. Haupt Y, Maya R, Kazaz A, Oren M. Mdm2 promotes the rapid degradation of p53. *Nature* 387, 296-299 (1997).
81. Mayo LD, Donner DB. A phosphatidylinositol 3-kinase/Akt pathway promotes translocation of Mdm2 from the cytoplasm to the nucleus. *Proc Natl Acad Sci U S A* 98, 11598-11603 (2001).
82. Ogawara Y*, et al.* Akt enhances Mdm2-mediated ubiquitination and degradation of p53. *J Biol Chem* 277, 21843-21850 (2002).
83. Maya R*, et al.* ATM-dependent phosphorylation of Mdm2 on serine 395: role in p53 activation by DNA damage. *Genes & development* 15, 1067-1077 (2001).
84. Gannon HS, Woda BA, Jones SN. ATM phosphorylation of Mdm2 Ser394 regulates the amplitude and duration of the DNA damage response in mice. *Cancer Cell* 21, 668-679 (2012).
85. Kulikov R, Boehme KA, Blattner C. Glycogen synthase kinase 3-dependent phosphorylation of Mdm2 regulates p53 abundance. *Mol Cell Biol* 25, 7170-7180 (2005).
86. Banin S*, et al.* Enhanced phosphorylation of p53 by ATM in response to DNA damage. *Science* 281, 1674-1677 (1998).
87. Guo Z, Kozlov S, Lavin MF, Person MD, Paull TT. ATM activation by oxidative stress. *Science* 330, 517-521 (2010).
88. Buschmann T*, et al.* Jun NH2-terminal kinase phosphorylation of p53 on Thr-81 is important for p53 stabilization and transcriptional activities in response to stress. *Mol Cell Biol* 21, 2743-2754 (2001).
89. Reyes-Zurita FJ, Pachon-Pena G, Lizarraga D, Rufino-Palomares EE, Cascante M, Lupianez JA. The natural triterpene maslinic acid induces apoptosis in HT29 colon cancer cells by a JNK-p53-dependent mechanism. *BMC Cancer* 11, 154 (2011).
90. Tang Y, Eng C. PTEN autoregulates its expression by stabilization of p53 in a phosphatase-independent manner. *Cancer Res* 66, 736-742 (2006).
91. Freeman DJ*, et al.* PTEN tumor suppressor regulates p53 protein levels and activity through phosphatase-dependent and -independent mechanisms. *Cancer Cell* 3, 117-130 (2003).
92. Bunz F*, et al.* Requirement for p53 and p21 to sustain G2 arrest after DNA damage. *Science* 282, 1497-1501 (1998).
93. Waldman T, Kinzler KW, Vogelstein B. p21 is necessary for the p53-mediated G1 arrest in human cancer cells. *Cancer Res* 55, 5187-5190 (1995).
94. Rossig L, Badorff C, Holzmann Y, Zeiher AM, Dimmeler S. Glycogen synthase kinase-3 couples AKT-dependent signaling to the regulation of p21Cip1 degradation. *J Biol Chem* 277, 9684-9689 (2002).
95. Lee JY, Yu SJ, Park YG, Kim J, Sohn J. Glycogen synthase kinase 3beta phosphorylates p21WAF1/CIP1 for proteasomal degradation after UV irradiation. *Mol Cell Biol* 27, 3187-3198 (2007).
96. Chai F, Evdokiou A, Young GP, Zalewski PD. Involvement of p21(Waf1/Cip1) and its cleavage by DEVD-caspase during apoptosis of colorectal cancer cells induced by butyrate. *Carcinogenesis* 21, 7-14 (2000).
97. Park JA, Kim KW, Kim SI, Lee SK. Caspase 3 specifically cleaves p21WAF1/CIP1 in the earlier stage of apoptosis in SK-HEP-1 human hepatoma cells. *Eur J Biochem* 257, 242-248 (1998).

98. Xu Y, Pasche B. TGF-beta signaling alterations and susceptibility to colorectal cancer. *Hum Mol Genet* 16 Spec No 1, R14-20 (2007).
99. Dennler S, Prunier C, Ferrand N, Gauthier JM, Atfi A. c-Jun inhibits transforming growth factor beta-mediated transcription by repressing Smad3 transcriptional activity. *J Biol Chem* 275, 28858-28865 (2000).
100. Nakao A*, et al.* Identification of Smad7, a TGFbeta-inducible antagonist of TGF-beta signalling. *Nature* 389, 631-635 (1997).
101. Monteleone G, Caruso R, Pallone F. Role of Smad7 in inflammatory bowel diseases. *World J Gastroenterol* 18, 5664-5668 (2012).
102. Bitzer M*, et al.* A mechanism of suppression of TGF-beta/SMAD signaling by NF-kappa B/RelA. *Genes & development* 14, 187-197 (2000).
103. Macatonia SE*, et al.* Dendritic cells produce IL-12 and direct the development of Th1 cells from naive CD4+ T cells. *J Immunol* 154, 5071-5079 (1995).
104. Schroder K, Hertzog PJ, Ravasi T, Hume DA. Interferon-gamma: an overview of signals, mechanisms and functions. *J Leukoc Biol* 75, 163-189 (2004).
105. Maraskovsky E, Chen WF, Shortman K. IL-2 and IFN-gamma are two necessary lymphokines in the development of cytolytic T cells. *J Immunol* 143, 1210-1214 (1989).
106. Waldner MJ, Neurath MF. Colitis-associated cancer: the role of T cells in tumor development. *Semin Immunopathol* 31, 249-256 (2009).
107. Maroof A*, et al.* Interleukin-4 can induce interleukin-4 production in dendritic cells. *Immunology* 117, 271-279 (2006).
108. O'Farrell AM, Liu Y, Moore KW, Mui AL. IL-10 inhibits macrophage activation and proliferation by distinct signaling mechanisms: evidence for Stat3-dependent and -independent pathways. *EMBO J* 17, 1006-1018 (1998).
109. Buelens C, Verhasselt V, De Groote D, Thielemans K, Goldman M, Willems F. Human dendritic cell responses to lipopolysaccharide and CD40 ligation are differentially regulated by interleukin-10. *Eur J Immunol* 27, 1848-1852 (1997).
110. Banchereau J, Pascual V, O'Garra A. From IL-2 to IL-37: the expanding spectrum of anti-inflammatory cytokines. *Nat Immunol* 13, 925-931 (2012).
111. Thomas DA, Massague J. TGF-beta directly targets cytotoxic T cell functions during tumor evasion of immune surveillance. *Cancer Cell* 8, 369-380 (2005).
112. Fujimoto M*, et al.* The influence of excessive IL-6 production in vivo on the development and function of Foxp3+ regulatory T cells. *J Immunol* 186, 32-40 (2011).
113. Auray G, Lacroix-Lamande S, Mancassola R, Dimier-Poisson I, Laurent F. Involvement of intestinal epithelial cells in dendritic cell recruitment during C. parvum infection. *Microbes Infect* 9, 574-582 (2007).
114. Sagar GH, Bellare JR. Estimation of mechanical strength of unilamellar and multilamellar AOT/water vesicles and their rupture using micropipet aspiration. *J Phys Chem B* 113, 13805-13810 (2009).
115. Um HD*, et al.* TNF-alpha suppresses dendritic cell death and the production of reactive oxygen intermediates induced by plasma withdrawal. *Exp Dermatol* 13, 282-288 (2004).
116. Brunner C*, et al.* Enhanced dendritic cell maturation by TNF-alpha or cytidine-phosphate-guanosine DNA drives T cell activation in vitro and therapeutic anti-tumor immune responses in vivo. *J Immunol* 165, 6278-6286 (2000).
